# No Evidence of Direct Transmission of Emerging Bluetongue Virus Strains Between Israel and Europe Based on Genomic Analyses (2013–2023)

**DOI:** 10.3390/pathogens15010038

**Published:** 2025-12-28

**Authors:** Natalia Golender, Eyal Klement, Bernd Hoffmann

**Affiliations:** 1 Department of Virology, Kimron Veterinary Institute, Bet Dagan 5025001, Israel; 2 Koret School of Veterinary Medicine, The Robert H. Smith Faculty of Agriculture, Food and Environment, The Hebrew University of Jerusalem, P.O. Box 12, Rehovot 7610001, Israel; eyal.klement@gmail.com; 3 Institute of Diagnostic Virology, Friedrich-Loeffler-Institut, Südufer 10, 17493 Greifswald-Insel Riems, Germany; bernd.hoffmann@fli.de

**Keywords:** *Reoviridae*, *Orbivirus*, bluetongue virus, cattle, sheep, ruminants, descriptive epidemiology, sequencing, phylogeny

## Abstract

Bluetongue (BT) is an arthropod-borne viral disease primarily affecting domestic and wild ruminants. In recent years, several BTV serotypes and genotypes have been detected in Israel almost annually, raising questions about their origin and routes of introduction. Some BTV serotypes closely related to those first identified in Israel, including BTV-3, BTV-8, and BTV-12, were subsequently reported in Europe after a delay of several years. In this study, we sequenced the complete genomes of one representative strain of all newly identified Israeli BTV genotypes/serotypes—BTV-1, -4, -5, -8, and -11—first detected between 2021 and 2023. Additionally, complete sequences of enzootic Israeli BTV (2015) and eleven BTV-3 strains (2019–2023), with two representative strains for every year of isolation, except 2021 (three strains), were analyzed using phylogenetic, BLAST, and pairwise identity approaches. Genetic analyses revealed that recently identified Israeli and European BTV strains share common African ancestors, with some genomic “incursions” from Mayotte Island or the Arabian Peninsula. These incursions appeared more frequently in Israeli than in European strains. Nevertheless, nucleotide sequence differences of at least 2–3% across all genes indicate several years of independent evolution. The observed divergence suggests that no direct transmission of BTV occurred between Israel and Europe during the past decade.

## 1. Introduction

Bluetongue (BT) is a widely distributed viral disease of domestic and wild ruminants caused by bluetongue virus (BTV) [1]. There is no public health risk associated with BT [2,3]. Since BT causes substantial economic and animal health impacts, the World Organization for Animal Health (WOAH) classifies BT as a notifiable disease of global concern [3].

Severe clinical manifestations, often resulting in acute death, are primarily observed in sheep and certain wildlife species, such as white-tailed deer. The course of BT in sheep can vary from peracute to chronic, with a mortality rate ranging from 2% to 90%. Peracutely affected animals may die within 7–9 days of infection, most often due to severe pulmonary edema leading to dyspnea, frothing from the nostrils, and death by asphyxiation [2].

Infection in cattle is uncommon but may resemble that observed in sheep [4]. The clinical disease is usually characterized by fever, increased respiratory rate, lacrimation, hypersalivation resulting from oral vesicles and ulcers, stiffness or lameness with inflammation at the junction of the skin and coronary band, hyperesthesia, vesicular and ulcerative dermatitis, ocular and nasal discharge, swelling of the mouth, head, and neck, respiratory distress, and abortion [5]. Infection in goats is generally subclinical or causes only mild clinical signs [6].

Economic losses caused by BT are associated with direct and indirect costs. Direct costs are included in production losses, which are mostly linked with mortality, morbidity, reduced milk yield, abortions, and reduced fertility rate. Indirect losses are estimated at the national level, which are associated with control and surveillance costs, trade restrictions, vaccination, diagnosis, and vector monitoring [7].

BTV is a member of the *Orbivirus* genus within the family *Sedoreoviridae*. It is characterized by a double-stranded RNA genome divided into ten segments of different sizes [8,9]. The BTV genome consists of 10 linear double-stranded RNA segments (Seg-1 to Seg-10) encoding seven structural (VP1–VP7) and five nonstructural (NS1–NS5) proteins [10]. Like other segmented viruses, BTV undergoes genetic reassortment during co-infections, allowing the exchange of genome segments between strains [11]. The BTV outer-capsid proteins VP2 and VP5, encoded by genome segments 2 and 6 (Seg-2 and Seg-6), respectively, play key roles in cell attachment and entry during the early stages of infection. These proteins—particularly VP2—also contain epitopes that bind neutralizing antibodies generated during infection of the mammalian host. Differentiation within serotypes and between different serotypes is now based on both classical methods (serum neutralization tests) and molecular approaches (sequence analysis) [12]. Currently, thirty-six distinct BTV serotypes have been officially recognized based on sequence differences in the Seg-2 gene and virus neutralization tests [13,14].

Transmission of most BTV serotypes between mammalian hosts relies on competent, blood-feeding midges of the *Culicoides* species [1]. Vertical and horizontal transmissions have been described and/or hypothesized, but they are considered to be of less epidemiological importance [15,16,17,18,19,20].

Officially, the history of BT in Europe begins with the identification of BTV-3 in Cyprus in 1943, although it was possibly introduced into the country earlier and clinically diagnosed as “stomatitis” as early as 1924 [21,22]. The extent and severity of BTV outbreaks in Cyprus apparently varied from year to year, with the most virulent occurring in 1924, 1939, 1943, 1951, 1965, and 1977 [23]. Before 1998, BTV caused only two outbreaks elsewhere in Europe: a major outbreak of BTV-10 on the Iberian Peninsula between 1956 and 1960 [24] and a smaller outbreak of BTV-4 on several Greek islands in the Aegean in 1979–1980 [25]. BTV-4, closely related to these Greek BTV-4 strains but differing in several internal genes, circulated in the Mediterranean Basin (Cyprus and Turkey) between 1979 and 2004 [26]. This situation changed dramatically from 1998, when BTV-9 was detected on several Greek islands and spread rapidly throughout the Mediterranean Basin (Turkey, Bulgaria, Serbia, Montenegro, Kosovo, and Macedonia) [27], followed by incursions of BTV-1, BTV-2, BTV-4, and BTV-16 during 2000–2005 in Italy, Turkey, Greece, Balkan countries, and France (Corsica) [23]. Since 2006, a BTV-1 strain of Algerian origin has expanded northwards across the Iberian Peninsula and France [28]. In August 2006, BTV crossed latitude 50° N for the first time, and BT outbreaks caused by BTV-8 occurred in the Netherlands, Belgium, Germany, France, and Luxembourg. In 2008, BTV-6 and BTV-11 were detected in Germany, the Netherlands, and Belgium [29,30]. On this occasion, mass vaccination campaigns implemented in Europe in the spring of 2008 quickly limited the spread of disease caused by BTV-8, and it was eradicated by 2011. However, after a three-year hiatus, in September 2015, BTV-8 re-emerged in central France and subsequently spread throughout the entire country. The renewed outbreak of this BTV-8 strain was most likely caused by the use of cryopreserved virus-positive semen or embryos. In the following years, BTV-8 outbreaks were reported in Switzerland, Germany, Belgium, and Spain [30,31,32].

In 2023, a new genetically distinct BTV-8 strain was also discovered in France [9]. In addition to BTV-8, serotypes 1, 2, 4, 9, and 16 have recently circulated in Europe [31]. The history of the current incursions of BTV-3 in Europe begins with its identification in Italy [33]. However, the spread of BTV-3 through continental Europe was registered in September 2023, when clinical disease was observed on four sheep farms in the Netherlands [34]. Cases of BTV-3 have since spread across 13 European countries (Netherlands, Belgium, Germany, UK, Denmark, France, Luxembourg, Switzerland, the Czech Republic, Portugal, Sweden, Austria, and Norway) [35].

Most recently, in October 2024, BTV-12 was detected in cattle and sheep in the Netherlands [36] and later in England (United Kingdom) [37].

**Figure 1:**
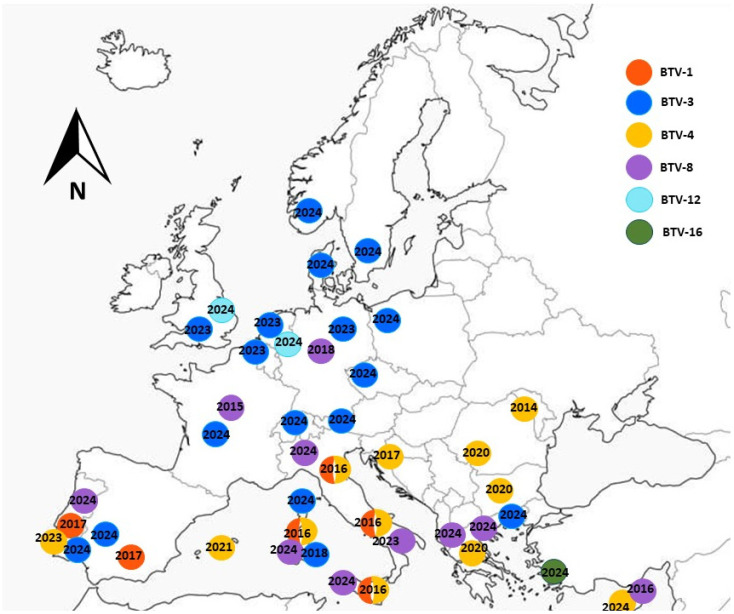
Bluetongue outbreaks in Europe recorded during 2015–2024.

A schematic map showing the first detection of each BTV serotype in Europe over the past decade is presented in Figure 1. The information was summarized based on recent publications and annual WOAH reports [31,33,38,39,40,41,42].

In Israel, BT was first observed in 1944. From 1964 to 2004, five serotypes were found to be circulating: BTV-2, -4, -6, -10, and -16 [43]. Between 2004 and 2020, thirteen BTV serotypes were detected in Israel—BTV-1, -2, -3, -4, -5, -6, -8, -9, -10, -12, -15, -16, and -24—with BTV-3 and BTV-4 remaining enzootic in recent years [44].

The increasing frequency of incursions by new BTV strains, along with the temporal similarity in the emergence of novel serotypes in Israel and Europe, raises questions about the origin and possible transmission routes of these viruses.

In the present study, recently identified Israeli BTV strains were sequenced and compared with publicly available BTV sequences. The objectives were to (i) describe the BTV strains detected in Israel between 2020 and 2023 and (ii) compare Israeli and European BTV strains using BLASTn (NCBI) [45], pairwise, and phylogenetic analyses, in order to identify shared and distinct patterns in the emergence of previously undetected BTV strains in both regions.

## 2. Materials and Methods

### 2.1. Field Samples

Samples from weak, dead, or aborted domestic and wild/zoo ruminants submitted between 2021 and 2023 for routine arboviral infection testing to the Virology Department of the Kimron Veterinary Institute (KVI), Israel, were included in this study. Clinical specimens included placenta, brain, and internal organs from aborted fetuses; whole blood from symptomatic animals; and spleen or lung tissue from dead ruminants.

### 2.2. Virus Isolation (VI)

Due to coronavirus restrictions and the increased number of field samples received during 2021–2023—associated with simultaneous outbreaks of several arboviral diseases in livestock caused by multiple serotypes of BTV, bovine ephemeral fever virus (BEFV) [46], and equine encephalosis virus serotype 6 (EEV-6; arboviral season of 2023, unpublished data)—only selected BTV RT-qPCR-positive samples collected between 2021 and 2023 were inoculated into embryonated chicken eggs (ECE; VALO BioMedia North America LLC) according to the method described by Komarov and Goldsmit [47]. In brief, infectious material was inoculated intravenously into 9–11-day-old ECE and incubated in an egg incubator at 35 °C. Infected ECE were monitored for seven days post-inoculation. Whole embryos, in which embryonic death occurred, were homogenized and tested using pan-BTV RT-qPCR to confirm VI. Some of the viruses isolated in ECE were subsequently adapted to Vero (African green monkey kidney epithelial cells; source- ATCC), BHK-21 (source- ATCC), or BHK-BSR cells (baby hamster kidney cells or their clone, BSR), kindly provided by Prof. Eran Bachrach, Tel Aviv University, Israel. The procedures for sample preparation for VI and the entire VI process are described in Golender et al.’s study [46].

### 2.3. Nucleic Acid Extraction and Pan-BTV Real-Time Polymerase Chain Reaction (RT-PCR)

Ribonucleic acid (RNA) was extracted from tissue culture supernatants, chicken embryo homogenates, and field samples (whole blood, lung, brain, spleen) using the MagMAX™ CORE Nucleic Acid Purification Kit (Thermo Fisher Scientific, Austin, TX, USA) or the IndiMag Pathogen Kit (Indical Bioscience, Leipzig, Germany), following the manufacturers’ instructions. Viral RNA detection was performed using the VetMAX™ BTV NS3 All Genotypes Kit (Applied Biosystems™, Thermo Fisher Scientific, Lissieu, France) or according to Wernike et al. [48], with amplification carried out using the AgPath-ID™ One-Step RT-PCR Kit (Life Technologies, Austin, TX, USA). Samples of cattle origin were additionally tested for epizootic hemorrhagic disease virus (EHDV) as described by Wernike et al. [48], and for BEFV following the protocols of Erster et al. [49] and Golender et al. [50]. According to the authors’ and manufacturers’ instructions for each RT-qPCR system, the cut-off value for all assays was a cycle threshold (Ct) of 40.

### 2.4. Type-Specific Real Time (RT-qPCR) and Conventional RT-PCRs

The method described by Lorusso et al. [51] was used for the detection of BTV-3. For the detection of BTV-1, BTV-4, and BTV-8 genomes, assays described by Maan et al. [52] were performed. During 2021–2023, samples that tested positive for BTV by RT-qPCR with Ct values ≤34 were further analyzed using BTV-3- and BTV-4-specific RT-qPCR assays, as these serotypes are currently enzootic in Israel.

Screening of pan-BTV positive samples by RT-qPCR for additional serotypes was performed retrospectively, based on the results of virus isolation and serotype determination by in-house RT-PCR, followed by confirmation using Sanger sequencing, as previously described [53]. Primers for the BTV-1-specific conventional RT-PCR are listed in the Supplementary Material (Table S1).

Due to the identification of a critical mutation in BTV-12 Israeli strains from 2020–2021 that affected the applicability of the assay described by Maan et al. [52], the oligonucleotide (nt) sequence of the probe was modified, tested for specificity, and used in a limited manner (Table S1). For this reason, only a limited number of BTV-positive samples collected during the early phase of the outbreak (June–July 2021) were tested using conventional BTV-12–specific RT-PCR and subsequently confirmed by Sanger sequencing [53]. Screening of BTV-positive samples with the modified BTV-12 RT-qPCR assay was conducted on field samples received in early 2022 (January–June 2022).

All RT-qPCR assays were performed using the AgPath-ID™ One-Step RT-PCR Kit (Life Technologies, Austin, TX, USA), and conventional RT-PCR assays were carried out using the OneStep RT-PCR Kit (Qiagen, Hilden, Germany).

### 2.5. Whole Genome Sequencing (WGS) and Phylogenetic Analyses

Every successfully isolated BTV strain was sequenced by Sanger sequencing using serotype-specific primers for final confirmation of the serotype [53]. Based on these results, each virus was assigned to a specific serotype/genotype.

We sequenced the complete genomes of one representative strain for each newly identified Israeli BTV genotype/serotype—BTV-1, -4, -5, -8, and -11—first detected between 2021 and 2023. Only one BTV-8 isolate was available, and the single BTV-4 strain had already been successfully adapted to tissue culture, which determined the choice of these strains. The choice of a specific strain among several belonging to the same serotype (BTV-1, -5, and -11) was based on the lower Ct values in RT-qPCR obtained for the tissue-culture–adapted strains.

Additionally, we sequenced complete genomes of enzootic Israeli BTV-4 (2015) and eleven BTV-3 strains isolated during 2019–2023. To better understand the dynamics of BTV evolution, and as a continuation of our previously published study on Israeli BTV-3 [53], two representative strains from each year were included, except for 2021 (three strains). Two different sub-genotypes were detected simultaneously in 2020, and one strain from each sub-genotype was sequenced. Sample preparation for WGS of six Israeli strains was performed at the Friedrich-Loeffler-Institut, Germany. High-throughput sequencing (HTS) was carried out using the sequence-independent single-primer amplification (SISPA) approach [54], following the procedure described by Ries et al. [55] for double-stranded cDNA preparation. The resulting cDNA was submitted to Eurofins Genomics (Ebersberg, Germany) for genome sequencing on the Illumina platform.

RNA extraction for 11 additional Israeli strains was performed at the KVI, Israel, using either the Invisorb Spin Virus RNA Mini Kit (STRATEC Molecular GmbH, Berlin, Germany) or the GeneAll RiboSpin™ vRD II Kit (GeneAll Biotechnology, Songpa-gu, Seoul, Korea). Extracted RNA was transferred to the Technion Genomic Center (Technion—Israel Institute of Technology, Haifa, Israel) for high-throughput sequencing using the method previously described by Golender et al. [56].

The resulting nucleotide (nt) sequences were assembled and aligned pairwise using Geneious version 9.0.5 (Biomatters, Auckland, New Zealand) and/or BioEdit (https://bioedit.software.informer.com/7.2/). Phylogenetic trees were constructed using MEGA X software [57]. For all phylogenetic analyses, the maximum likelihood (ML) method and the Tamura–Nei model were applied.

## 3. Results

### 3.1. Field Samples

As described in our previous investigations, the most suitable materials for molecular detection and virus isolation are whole blood from sick animals, and spleen from dead animals, as well as spleen, brain, and placenta from aborted fetuses. Overall, the proportion of positive samples is highest in blood samples [44,53]. A detailed analysis of the sample types used in the current study is not provided.

A total of 4640 field samples were tested between 2021 and 2023. The results are summarized in Table 1. Field samples were systematically tested for serotypes 3 and 4, while for other serotypes, samples were tested partially. During 2021–2023, BTV-3 and -4 were constantly identified. In general, during 2021 and 2023, an increased number of samples were submitted to the Department of Virology, KVI, likely associated with simultaneous outbreaks of BTV and BEFV in those years. The total number of tested samples was similar in 2021 (*n* = 1899) and 2023 (*n* = 1868) (Table 1), while the number of BEFV-positive samples was also comparable: 526 in 2021 (according to the annual reports of the Veterinary Services and the KVI) and 506 in 2023 [46]. The proportion of BTV-positive samples in 2021 was significantly higher (*n* = 629; 33.12%) than in 2023 (*n* = 360; 19.26%). The increased number of BTV-positive samples in 2021 reflected more severe outbreaks, which caused prominent clinical signs in affected animals and led veterinary clinicians and animal owners to submit more samples for arboviral diagnostics. Thirteen of the eighty-eight samples tested positive, indicating the continued circulation of BTV-12 at the end of 2021 (Table 1).

### 3.2. Virus Isolation (VI)

Data on VI during 2021–2023 are presented in Table 2. During this period, eight serotypes were isolated from field samples (Table 2). Throughout the entire study period, BTV-3 and BTV-4 were consistently isolated. Since BTV-12 generally caused a low viral load in the bloodstream of infected cattle (most BTV-positive cattle blood samples had pan-BTV RT-qPCR Ct values above 30), virus isolation was often unsuccessful.

### 3.3. Summarized Data on Identified BTV Serotypes and Genotypes During 2013–2023

Detailed information on BTV identification during the past decade was described previously [44,53,58]. During 2021, five BTV serotypes were detected during the arboviral season: two enzootic strains (BTV-3 and BTV-4), BTV-11 (previously unreported in the region), a new genotype of BTV-12 that was first identified in Israel at the end of 2020, and a new genotype of BTV-1 (detailed genetic analyses of all Israeli BTV strains are presented in Section 3.5).

The BTV-1 strain identified at the end of 2021 continued to circulate in 2022 and became the dominant serotype that year. Additionally, BTV-3 and BTV-4 were identified and isolated (Table 1 and Table 2). In 2023, five serotypes were detected: BTV-3, BTV-4, BTV-5, BTV-6, and BTV-8. Three new genotypes were isolated that year, BTV-4, BTV-5, and BTV-8, although the current BTV-8 genotype was first molecularly detected in Israel in 2019 (Table 1 and Table 2).

**Figure 2:**
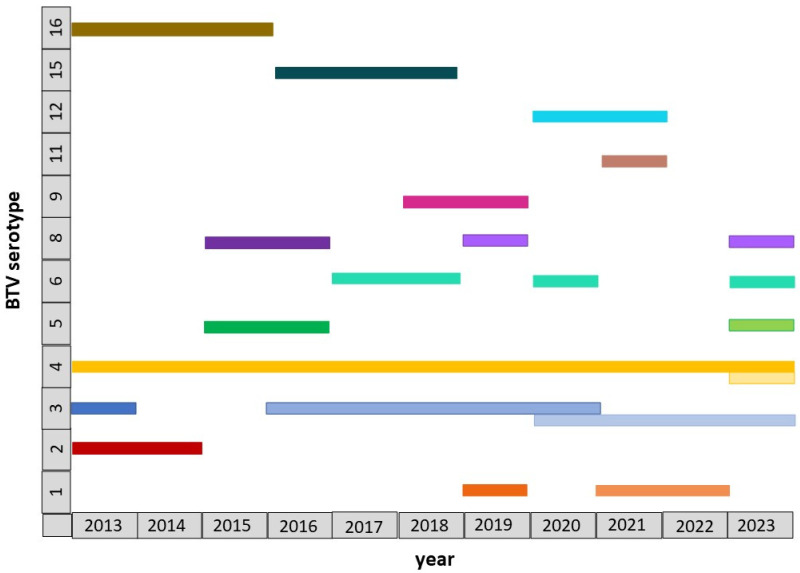
Schematic data on the BTV serotypes and genotypes identified in Israel during 2013–2023. BTV genotypes belonging to the same serotype are marked in the same color, with different shades used to distinguish between them.

Summarized information on BTV identification in Israel during 2013–2023 is presented in Figure 2.

### 3.4. Sequencing

Information about selected strains for sequencing is presented in Table 3. The increased number of completely sequenced Israeli BTV-3 strains can be attributed to the detection of several genetically distinct, non-ancestral BTV-3 genotypes, as determined by Sanger sequencing, suggesting multiple independent introduction events into Israel. Information about selected strains for sequencing is presented in Table 3.

### 3.5. Phylogenetic, BLAST, and Pairwise Analyses

#### 3.5.1. Analysis of Serotype /Genotype Defining Outer Proteins’ Coding Genes (Seg-2 and Seg-6)

- BTV-1

**Figure 3:**
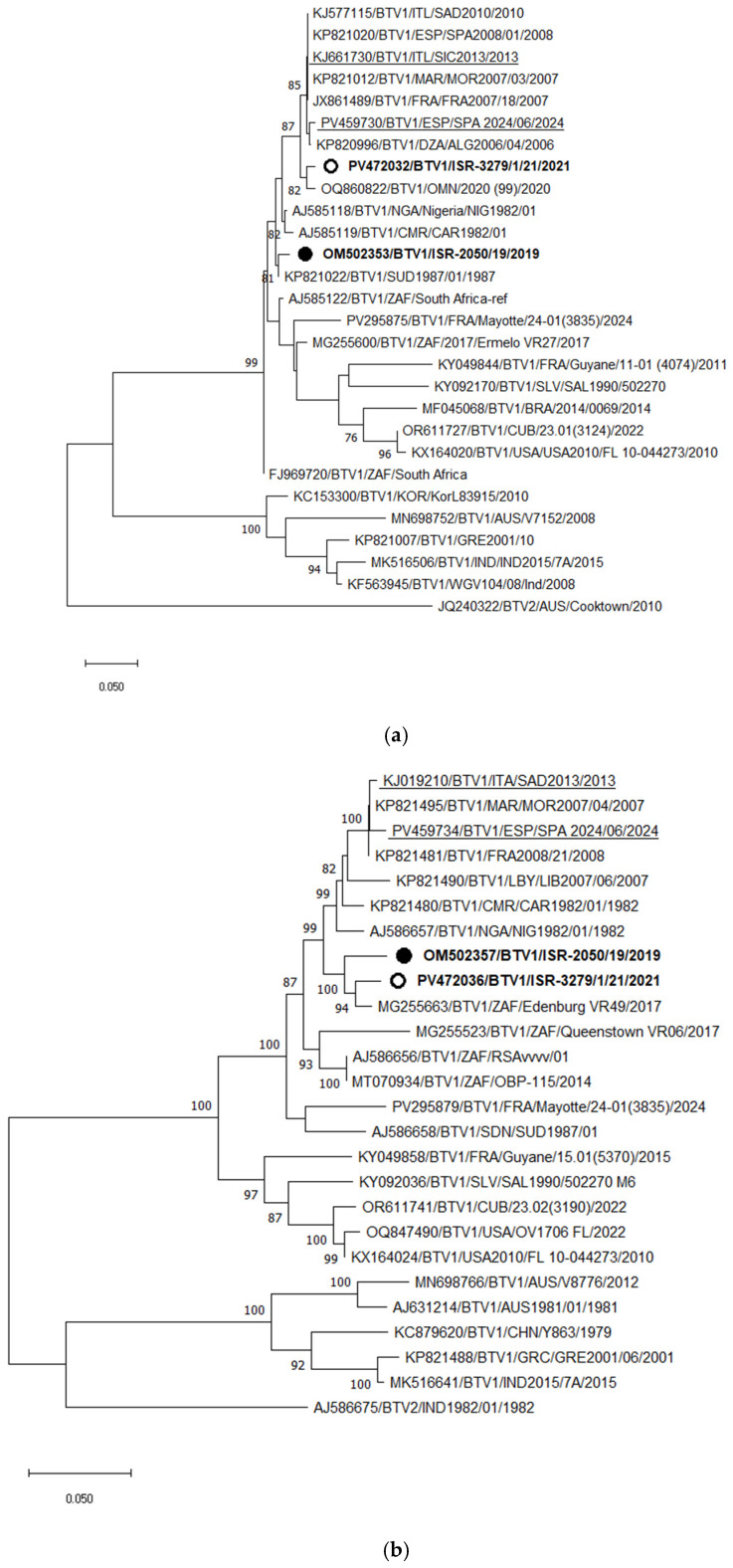
Phylogenetic tree of segment 2 (**a**) and segment 6 (**b**) of Israeli BTV-1 isolated in 2021 and global BTV-1 strains. BTV-2 was used as an outgroup. Israeli BTV-1 strains are shown in bold. The strain from 2019 is marked with a black circle; the strain from 2021 is shown with an empty circle. Recent European BTV-1 strains are highlighted. The phylogeny was inferred using the maximum likelihood method and the Tamura–Nei model. Statistical support for nodes was obtained by bootstrapping (1000 replicates); only values ≥ 70% are shown. Scale bars indicate nucleotide substitutions per site. Viruses were identified by accession number/serotype/location/isolate/year.

Phylogenetic analysis of Segment 2 (Seg-2) indicated that the Israeli BTV-1 strain ISR-3279/1/21, isolated in 2021, clustered with a BTV-1 strain from Oman identified in 2020, sharing 98.25% nt identity. These two strains also exhibited high similarity to BTV-1 strains circulating in North African and Southern European countries since 2006, with 97.66–98.25% nt identity. A new BTV-1 strain into Israel, which is markedly distinct from the BTV-1 strain previously identified in 2019, that clustered with a BTV-1 strain from Sudan (Figure 3a). Based on Seg-2 sequences, Israeli BTV-1 strains from 2019 and 2021 shared only 93.15% nt identity.

Phylogenetic analysis of Seg-6 showed that both Israeli BTV-1 strains from 2019 and 2021 clustered with a South African strain isolated in 2017 (Edinburgh VR49). The European BTV-1 strains formed a more distant Seg-6 clade relative to both genotypes of Israeli BTV-1 strains, as would be expected based on Seg-2 (Figure 3b). Since Seg-6 data are not available for the BTV-1 strain from Oman, its relationship to the Israeli viruses could not be assessed. Among the European strains, recently identified viruses clustered with strains from the Mediterranean Basin isolated approximately 1.5 decades ago, suggesting continuous circulation of these strains in southern Europe.

- BTV-3

**Figure 4:**
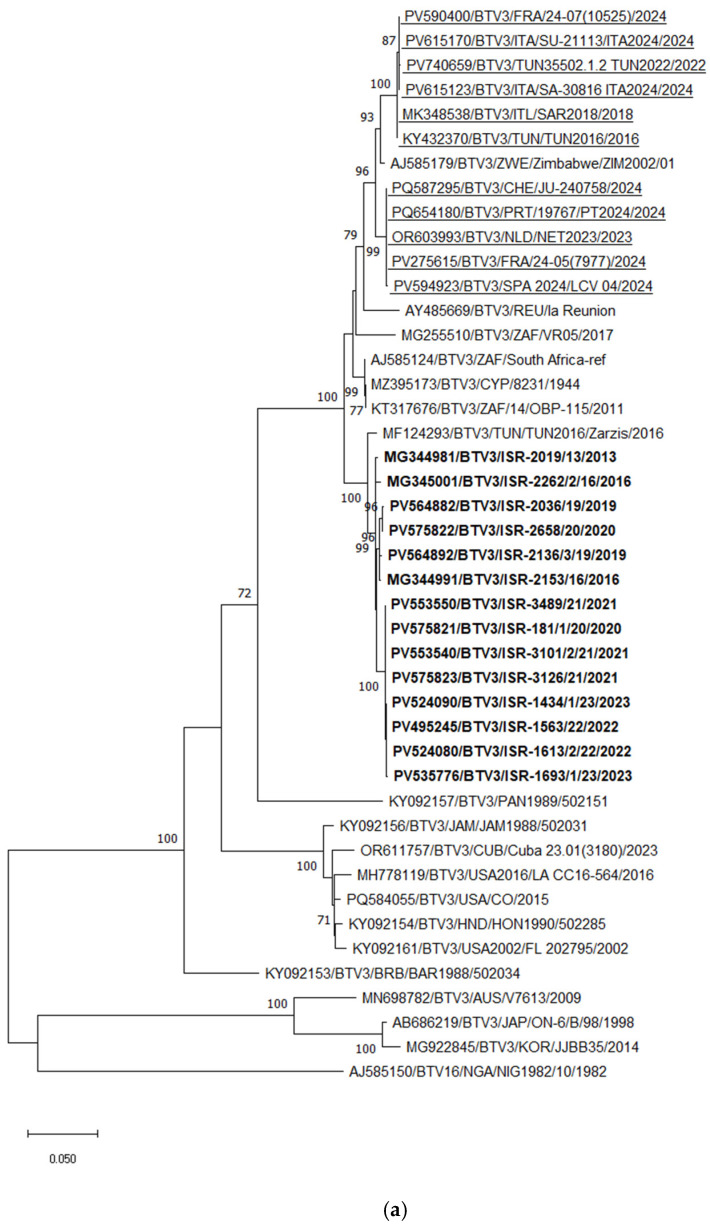

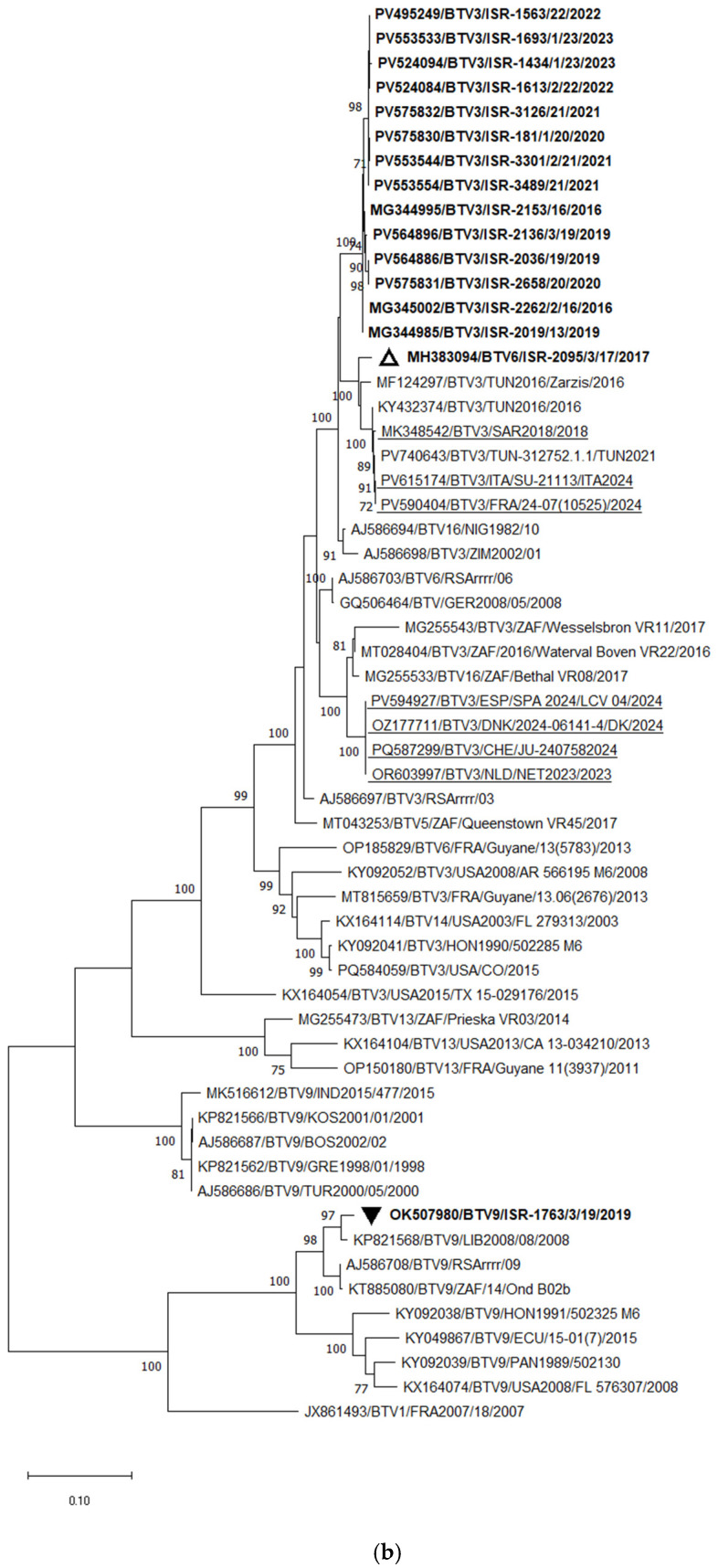
Phylogenetic tree of segment 2 and segment 6 of Israeli BTV-3 isolated between 2013 and 2023 and global BTV-3 strains: (**a**) Phylogenetic tree of segment 2 of BTV-3 and global BTV-3 strains. BTV-16 was used as an outgroup. Recently identified European BTV-3 (2018–2024) strains are highlighted. (**b**) Phylogenetic tree of BTV-3, -6, and -9 segment 6 strains. BTV-1 was used as an outgroup. Israeli BTV-3 strains are shown in bold. The Israeli BTV-6 strain is marked in bold and an empty triangle; Israeli BTV-9 is shown in bold and a black inverted triangle. The phylogeny was inferred using the maximum likelihood method and the Tamura–Nei model. Statistical support for nodes was obtained by bootstrapping (1000 replicates); only values ≥ 70% are shown. Scale bars indicate nucleotide substitutions per site. Viruses were identified by accession number/serotype/location/isolate/year.

Phylogenetic analysis of Seg-2 demonstrated that the Tunisian BTV-3 strain TUN2016/Zarsis is the closest relative of all Israeli BTV-3 strains. However, a more detailed analysis revealed the presence of several distinct subclusters among the Israeli strains. Two strains, ISR-2019/13 (isolated in 2013) and ISR-2262/2/16 (isolated in 2016), were more distantly related to the most recent Israeli BTV-3 strains. As previously reported [53], BTV-3 strains circulating between 2016 and 2020 were extremely closely related, sharing 99.59–99.62% nucleotide identity, and likely represent ancestral lineages. The BTV-3 strains circulating in Israel from 2020 to 2023 formed an additional subcluster, sharing 98.87–99.28% nt identity with the earlier strains (2016–2020) based on Seg-2 (Figure 4a). In comparison, both recently emerging European BTV-3 variants—Mediterranean and Central European types detected in 2023—were closely related to each other, sharing 97.51–97.55% nt identity, and clustered with the Zimbabwean strain ZIM2002/01. Pairwise comparison of the European (both variants) and Israeli BTV-3 strains revealed 93.92–93.96% nt identity.

BLASTn and phylogenetic analyses of Seg-6 revealed that BTV-3 sequences were closely related to Seg-6 sequences of BTV-6, -9, -13, -14, and -16. According to these analyses, reassortment events most frequently occurred between BTV-3, -6, and -16. Phylogenetic analysis of Seg-6 of Israeli BTV-3 strains showed results consistent with those obtained for Seg-2. All Israeli BTV-3 isolates clustered together, sharing 99.08–99.87% nt identity. Similar to Seg-2, strains from 2016–2020 formed a distinct subcluster, while strains from 2020–2023 formed another separate subcluster (Figure 4b).

Considering the European BTV-3 strains, the Italian isolates obtained between 2018 and 2024 clustered with Tunisian BTV-3 strains detected between 2016 and 2021, sharing 99.63–99.88% nt identity. These Italian strains also showed 97.18–97.49% nt identity with the Israeli BTV-6 strain ISR-2050/1/19 (2019) and 97.68–97.98% nt identity with the Tunisian BTV-3 strain TUN2016/Zarsis. According to phylogenetic analysis, the European BTV-3 strains exhibited 94.45–95.26% nt identity with the Israeli BTV-3 strains. Notably, the Seg-6 sequences of the BTV-3 strains recently detected in Central Europe (2023–2024) clustered with South African BTV-3 and BTV-16 strains, showing 96.88–97.19% nt identity (Figure 4b).

- BTV-4 and BTV-11

Phylogenetic analysis of Seg-2 showed that Israeli BTV-4 strains are highly similar to each other and, since 2001, have formed a distinct genetic cluster, sharing 98.97–99.45% nt identity. The strain that emerged in 2023 (ISR-1621/23) clustered with a Sudanese BTV-4 strain (96.21% nt identity), but shared only 93.04–93.18% identity with previously circulating Israeli BTV-4 strains.

**Figure 5:**
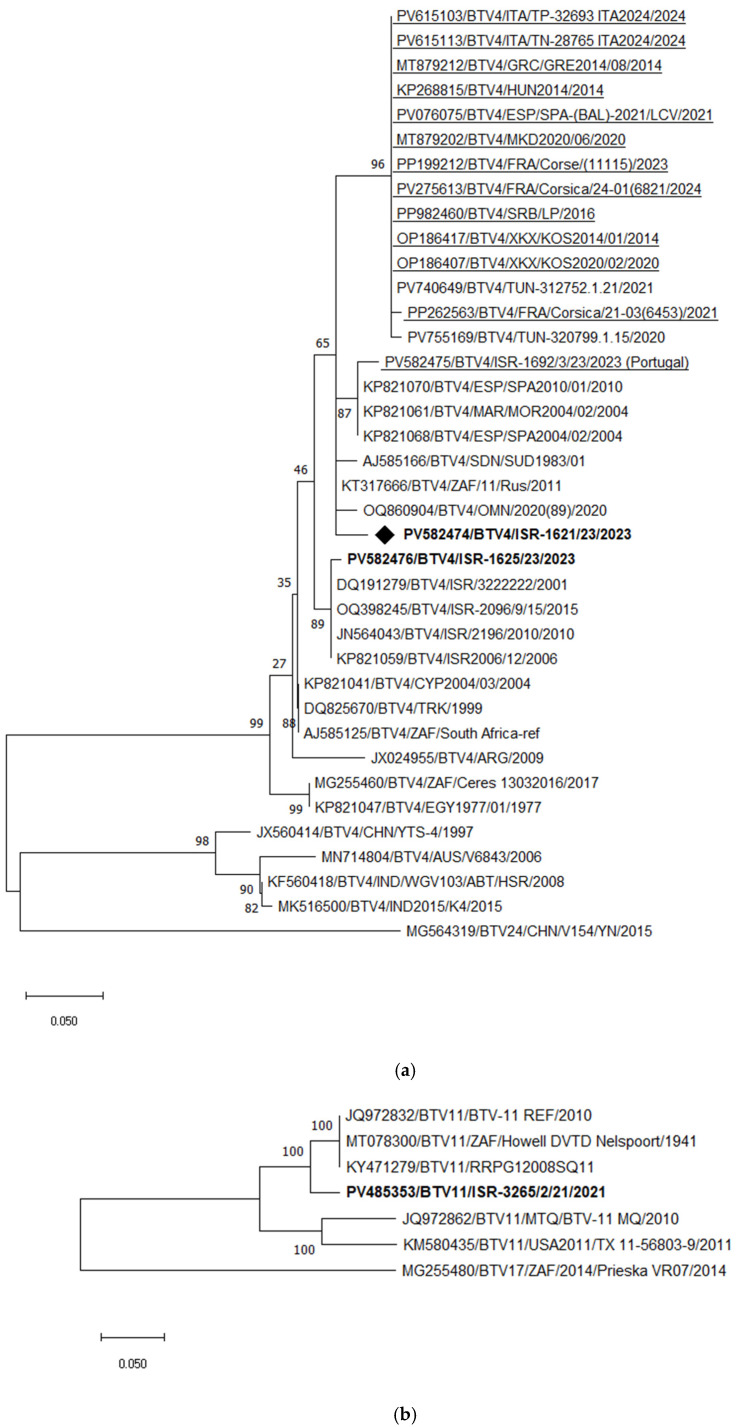

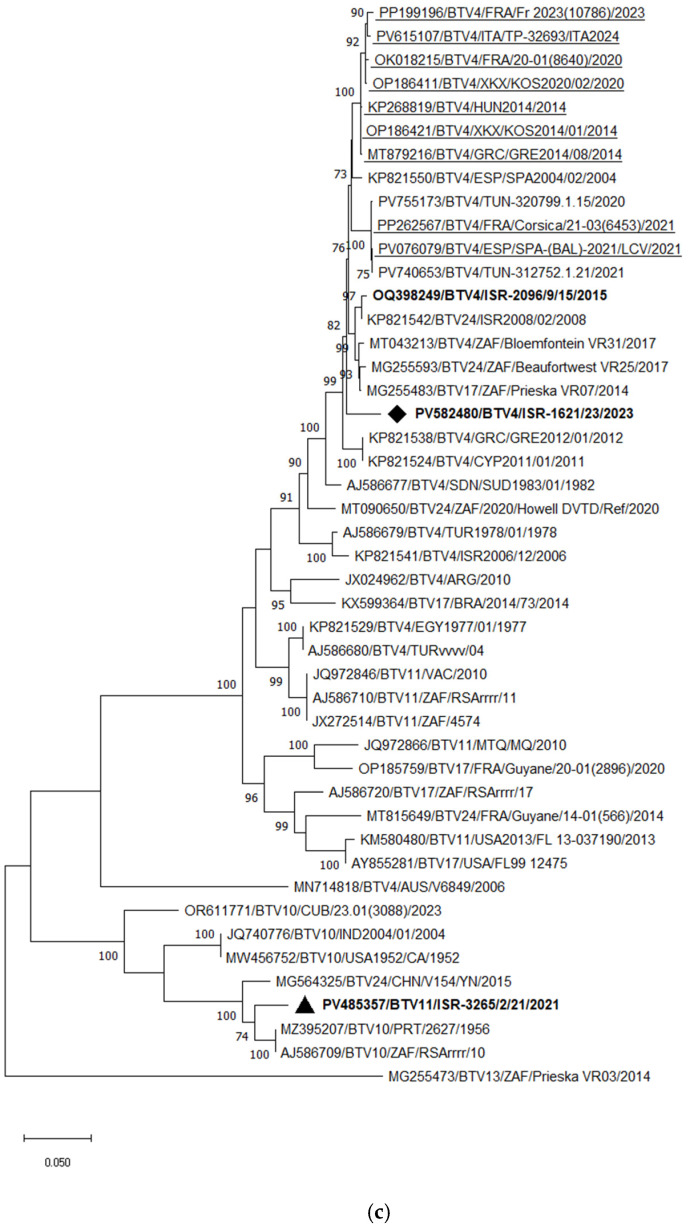
Phylogenetic tree of segment 2 and segment 6 of Israeli BTV-4 and 11 and global strains. (**a**) Phylogenetic tree of segment 2 of Israeli BTV-4 and global BTV-4 strains. BTV-24 was used as an outgroup. Recently identified European BTV-4 (2014–2024) strains are highlighted. Enzootic Israeli BTV-4 are marked in bold. Emerging Israeli BTV-4 is shown as a rhombus. (**b**) Phylogenetic tree of segment 2 of Israeli and global BTV-11 strains. BTV-17 was used as an outgroup. Israeli BTV-11 strains are shown in bold. (**c**) Phylogenetic tree of segment 6 of BTV-4 and BTV-11 strains. BTV-13 was used as an outgroup. The recent Israeli BTV-4 is shown in bold. The newly emerged Israeli BTV-4 strain is marked in bold and with a rhombus; Israeli BTV-11 is marked in bold and with a black triangle. The phylogeny was inferred using the maximum likelihood method and the Tamura–Nei model. Statistical support for nodes was obtained by bootstrapping (1000 replicates); only values ≥ 70% are shown. Scale bars indicate nucleotide substitutions per site. Viruses were identified by accession number/serotype/location/isolate/year.

At least three distinct BTV-4 genotypes are currently circulating in Europe. In 2023, a BTV-4 strain was identified in Israel from calves imported from Portugal [59]. Its partial Seg-2 sequence (ISR-1692/3/23) showed close relatedness (97.97% nt identity) to BTV-4 strains that circulated in North Africa and Spain between 2006 and 2010. Comparison of European BTV-4 strains circulating since 2014 with those from the Mediterranean region (Italy, Spain, France) revealed Seg-2 identities of 97.64–97.97%, and phylogenetic analysis grouped them within a single major cluster. However, when divergent BTV-4 and outgroup BTV-24 strains were excluded, European, Middle Eastern, and African strains separated into two closely related subclusters (Figure S1). In contrast, the Portuguese BTV-4 strain isolated in Israel (ISR-1692/3/23) and its related Spanish and Moroccan strains (2004–2010) exhibited lower identity (90.87–94.22%) with other European BTV-4 strains, forming a distinct lineage within the broader BTV-4 cluster (Figure 5a).

Phylogenetic analysis of Seg-2 demonstrated that the recently emerged Israeli BTV-11 strain clustered with South African strains, sharing 95.52–95.59% nt identity (Figure 5b).

Phylogenetic analysis of Seg-6, including the newly identified Israeli BTV-4 and BTV-11 strains, revealed that the 2023 BTV-4 strain (ISR-1621/23) formed a distinct monophyletic branch. BLASTn analysis showed its closest relatedness to South African BTV-17 (Prieska_VR07_2014) and Israeli BTV-24 (ISR2008/02), with 96.56% nt identity to both. Notably, Israeli BTV-4 strains circulating until 2006 clustered with a Turkish BTV-4 strain isolated in 1978 (Figure 5c), indicating a long-standing local lineage. Since the emergence of BTV-24 in Israel in 2008, local BTV-4 strains appear to have incorporated Seg-6 from BTV-24, as all subsequent local BTV-4 strains have consistently contained BTV-24–like Seg-6 sequences.

The emerging Israeli BTV-11 strain (ISR-3265/2/23) clustered with South African and Portuguese BTV-10 strains, sharing 95.98% nt identity (Figure 5c). Among European BTV-4 strains, two main clusters were identified: the first includes strains circulating in continental Europe since 2014 (notably Italian and French strains) with 99.08–99.63% nt identity, while the second includes Mediterranean strains from 2020–2021, sharing 99.74–99.87% identity. Unfortunately, the Seg-6 sequence of the “Portuguese” BTV-4 strain isolated in Israel was not available for analysis.

- BTV-5

**Figure 6:**
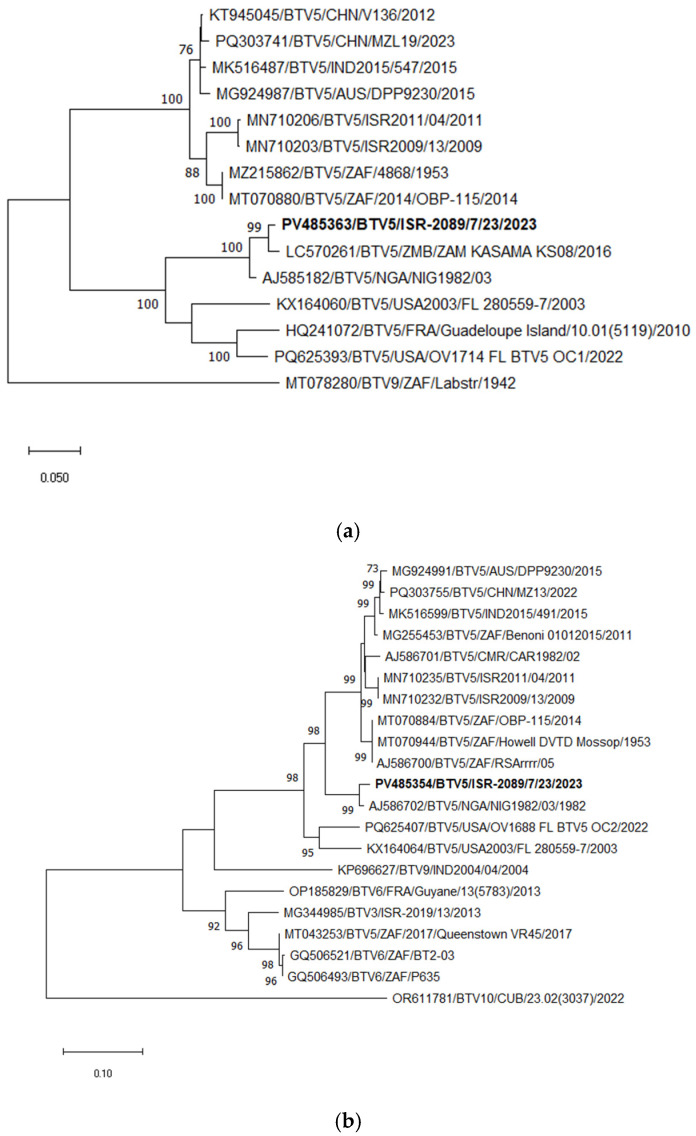
Phylogenetic tree of segment 2 and segment 6 of Israeli BTV-5 and relevant global strains: (**a**) Phylogenetic tree of segment 2 of Israeli BTV-5 and global BTV-5 strains. BTV-9 was used as an outgroup. Israeli BTV-5 is shown in bold. (**b**) Phylogenetic tree of segment 6 of Israeli and global BTV-5 and some other relevant strains. BTV-10 was used as an outgroup. Israeli BTV-5 is marked in bold. The phylogeny was inferred using the maximum likelihood method and the Tamura–Nei model. Statistical support for nodes was obtained by bootstrapping (1000 replicates); only values ≥70% are shown. Scale bars indicate nucleotide substitutions per site. Viruses were identified by accession number/serotype/location/isolate/year.

According to phylogenetic, BLASTn, and pairwise analyses of Seg-2, the Israeli BTV-5 strain ISR-2089/7/23 showed the closest relationship with the Zambian strain ZAM KASAMA KS08, sharing 98.18% nt identity, followed by the Nigerian strain NIG1982/03, with 96.82% nt identity. In contrast, comparison with previously circulating Israeli BTV-5 strains (detected between 2006 and 2016; available sequences ISR2009/13 and ISR2011/04) revealed only 78.07–78.32% nt identity (Figure 6a).

Phylogenetic, BLASTn, and pairwise analyses of Seg-6 of the newly emerged Israeli BTV-5 strain (ISR-2089/7/23) indicated the closest identity with the Nigerian BTV-5 strain NIG1982/03, sharing 98.11% nt identity (Seg-6 data for the Zambian strain ZAM KASAMA KS08 was not available). In contrast, the nt identity between ISR-2089/7/23 and previously circulating Israeli BTV-5 strains (2006–2016) was only 89.96–90.10% (Figure 6b).

- BTV-8

Phylogenetic analysis of Seg-2 illustrated the presence of two distinct genotypes of BTV-8 in Israel. The first genotype, detected between 2008 and 2019 (and responsible for outbreaks in 2010 and 2015–2016), was closely related to the European BTV-8 strains circulating since 2006 (“old strains”) [58]. The second, “new” genotype was first identified in Israel in 2019 [58] and re-detected in 2023. This new genotype forms a distinct monophyletic cluster within BTV-8.

**Figure 7:**
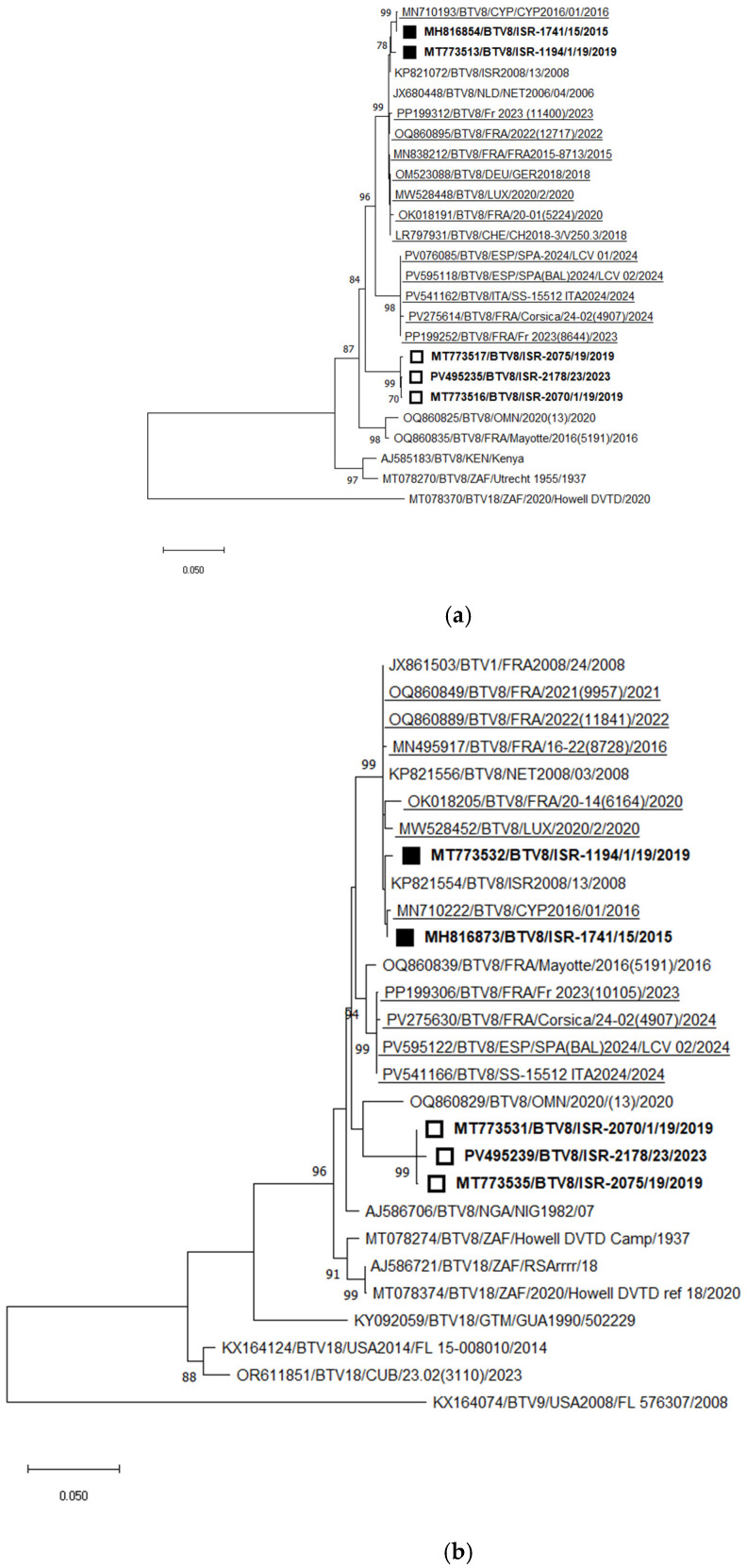
Phylogenetic tree of segment 2 and segment 6 of Israeli BTV-8 and global strains: (**a**) Phylogenetic tree of segment 2 of Israeli BTV-8 and global BTV-8 strains. BTV-18 was used as an outgroup. Recently identified European BTV-8 (2016–2024) are underlined. Recently emerging Israeli BTV-8 (2019–2023) are shown as an empty square; Israeli BTV-8 detected in 2015–2019 are shown as a black square. (**b**) Phylogenetic tree of segment 6 of Israeli and global BTV-8 strains. BTV-9 was used as an outgroup. Recently identified European BTV-4 (2014–2024) strains are highlighted. Recently emerging Israeli BTV-8 (2019–2023) are shown as an empty square; Israeli BTV-8 detected in 2015–2019 are shown with a black square. The phylogeny was inferred using the maximum likelihood method and the Tamura–Nei model. Statistical support for nodes was obtained by bootstrapping (1000 replicates); only values ≥ 70% are shown. Scale bars indicate nucleotide substitutions per site. Viruses were identified by accession number/serotype/location/isolate/year.

Among currently circulating European BTV-8 strains, two separate genotypes have been identified: one circulating in continental Europe and another spreading across Mediterranean Europe. The nt identity between these two European genotypes is 96.42–96.70%. The Israeli “new” BTV-8 genotype shares 94.89–94.99% nt identity with the “Mediterranean” European strains, and 95.34–95.61% nt identity with the “old” BTV-8 strains (both Israeli and European) (Figure 7a).

Phylogenetic analysis of Seg-6 of recently circulating BTV-8 strains in Eurasia revealed results consistent with the Seg-2 phylogeny. The only exception was that the Israeli “new” strains clustered with a BTV-8 strain from Oman (2020), sharing 95.60% nt identity. Similar to the Seg-2 phylogenetic analysis, the closest phylogenetic and pairwise nt identity for Seg-6 was observed between the “old” and “new” European strains (97.49–97.62%). The new Israeli BTV-8 strains formed a distinct monophyletic cluster.

Comparison of the “new” Israeli strains with the European “Mediterranean” and “old” genotypes showed nt identities of 94.86–94.93% and 95.41–95.60%, respectively (Figure 5b). Interestingly, the new Israeli BTV-8 strains clustered with the Omani BTV-8 strain, while BLASTn analysis indicated their closest identity with the Nigerian strain NIG1982/07, sharing 95.32–95.91% nt identity. The “Mediterranean” European BTV-8 strains clustered with the BTV-8 strain from Mayotte (2016; strain 5191), which was confirmed by BLASTn analysis, sharing 98.84–98.96% nt identity (Figure 7b).

- BTV-12

**Figure 8:**
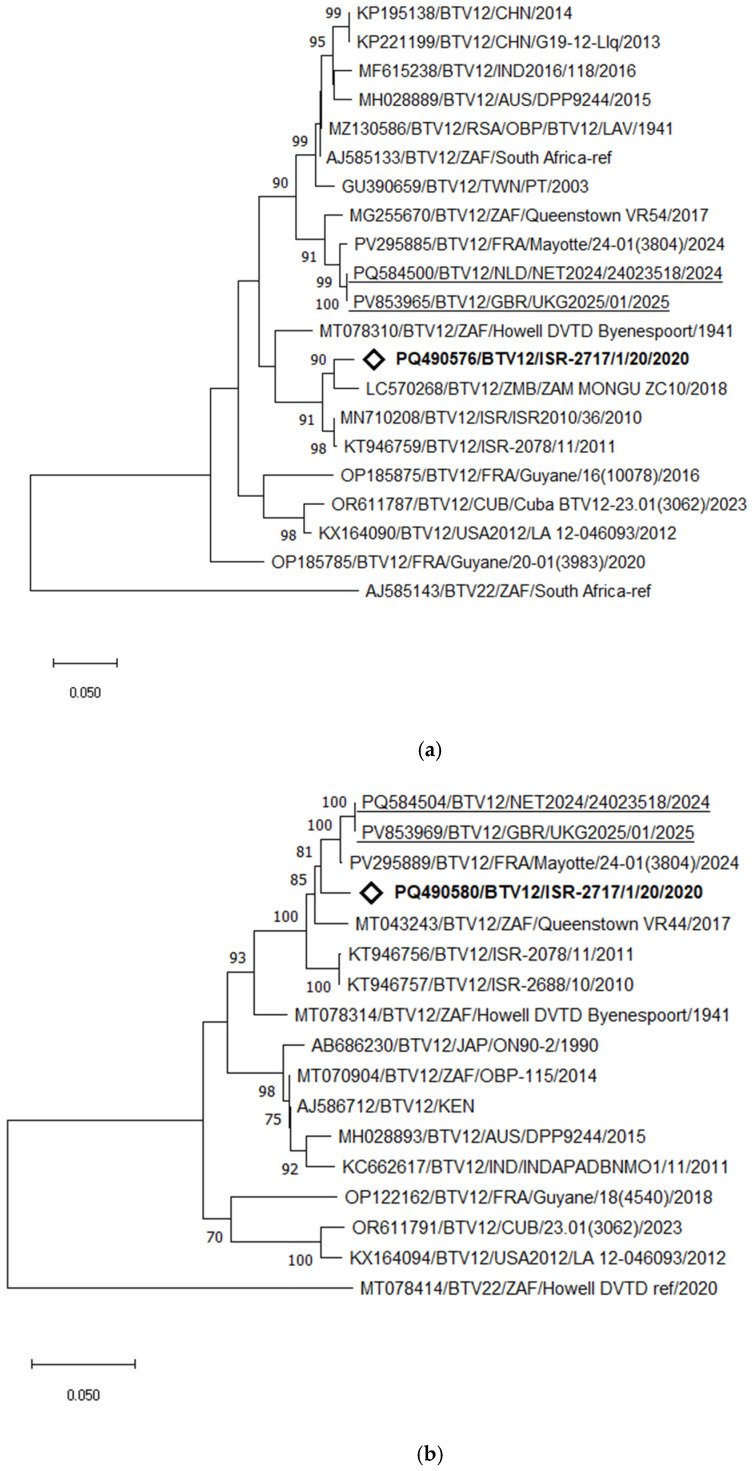
Phylogenetic tree of segment 2 (**a**) and segment 6 (**b**) of Israeli BTV-12 and global strains. Recently identified European BTV-12 strains are highlighted. The newly emerged Israeli BTV-12 strain is marked in bold and with the empty rhombus.. BTV-22 was used as an outgroup. The phylogeny was inferred using the maximum likelihood method and the Tamura–Nei model. Statistical support for nodes was obtained by bootstrapping (1000 replicates); only values ≥ 70% are shown. Scale bars indicate nucleotide substitutions per site. Viruses were identified by accession number/serotype/location/isolate/year.

Phylogenetic and pairwise analyses of the Israeli BTV-12 strain revealed its close relationship and clustering with the Zambian strain ZAM MONGU ZC10 (2018), sharing 96.73% nt identity. Pairwise comparison of the current Israeli BTV-12 strain with the earlier Israeli BTV-12 genotype detected in 2010–2011 showed 95.96–96.42% nt identity. In contrast, the recently detected BTV-12 strain in the Netherlands clustered with the strain from Mayotte Island, sharing 98.56% nt identity, followed by the South African strain isolated in 2017, which showed 96.56% nt identity with the strain from the Netherlands (Figure 8a).

Interestingly, phylogenetic analysis of Seg-6 revealed a notably closer relationship between the Israeli BTV-12 strain ISR-2717/1/20 and the recently identified BTV-12 strains from the Netherlands (NET2024/24023518) and Mayotte Island (strain 24–01(3804), 2024). The Israeli strain shared 97.52% nt identity with the Mayotte strain and 96.96% with the strain from the Netherlands. Notably, the strain from the Netherlands and Mayotte strains exhibited a very high nt identity of 99.21% (Figure 8b).

#### 3.5.2. Phylogenetic, BLASTn, and Pairwise Analyses of Internal Genes (Seg-1, -3, -4, -5, -7, -8, -9, and -10)

Detailed genetic analyses are presented in Supplementary Table S2 and Figure S2. In brief, BLASTn, pairwise, and phylogenetic analyses indicated that the majority of internal genes of the currently circulating Israeli strains are of local origin (see the total number of probable reassortment events in Supplementary Table S2). Most genes displayed a very high nt identity (typically >99%) with previously circulating Israeli strains, followed by strains of African origin. Notably, sequence similarity with strains from the Arabian Peninsula was minimal.

Interestingly, the recently identified BTV-3, BTV-4, and BTV-12 strains appear to share a probable common ancestor with some older Israeli strains, suggesting a shared evolutionary origin. However, the relatively low nt identity values exclude the possibility of direct viral transfer between these geographic regions, with the exception of Seg-7 of BTV-12, which showed 99.35% nt identity.

Considering BTV-12 cases in Israel and Europe, the currently identified BTV-12 strain from Mayotte Island exhibited close identity with both European and Israeli strains. In contrast, the BTV-4 strain isolated in 2015 (the oldest isolate analyzed in this study) showed higher identity with locally circulating strains from the pre-2015 period than with strains currently circulating in Israel.

## 4. Discussion

Israel is situated in a unique geographic location at the junction of three continents. Its close proximity to Africa, Asia, and Europe facilitates the potential introduction and spread of arboviral infections from all these directions. Historically, Israel has been endemic for BTV since the beginning of monitoring efforts [43].

During the past decade, numerous arboviruses belonging to the families *Sedoreoviridae*, *Peribunyaviridae*, and *Rhabdoviridae*, which had not been previously detected in Israel, have been identified and isolated on an annual basis [60]. Most of the identified simbuviruses showed close genetic relationships with African strains, with the exception of the most recent Akabane virus (AKAV) strains detected in 2018, which exhibited high identity with Turkish AKAV [60]. Interestingly, in 2023, a “Mayotte-like” BEFV strain was identified in Israel, whereas only local strains had been detected for more than two decades, with the exception of a transient introduction of a “Turkish” BEFV strain in 2008 [46].

Regarding other non-BTV orbiviruses, during the past decade, EHDV-1, -6, and -7 have been detected in Israel, with EHDV-1 and -6 likely originating from Africa [59]. Notably, this virus was previously detected in North African countries in 2006 and in Turkey in 2007, before reaching Israel in 2016—indicating a delay of approximately ten years and suggesting a natural mode of spread [59]. Furthermore, in 2023, an EEV-6 strain was identified and isolated in Israel, causing a severe outbreak in horses (unpublished data), which most likely also originated from Africa. 

The most recent orbiviral outbreaks, caused by EHDV-8, spread from the North African Mediterranean region into large parts of Europe. This virus first emerged in Tunisia in 2021, also pointing on African origin of the virus. Further EHDV-8 was subsequently identified in Italy and Spain the following year [61]. In 2023, EHDV-8 spread rapidly across Spain, Portugal, and France, and outbreaks were still being reported in 2024 in Spain, France, Portugal, and Andorra [62]. Historically, until 1998, the emergence and spread of previously unrecognized BTV strains were reported predominantly within the Mediterranean region. For example, the BTV-1 strain detected in Greece in 2001 was genetically similar to isolates originating from the Far East [63]. Another distinct BTV-1 strain emerged for the first time in Algeria in 2006 [64] and subsequently disseminated across the southern Mediterranean basin, spreading northward to France and even posing a potential threat to the United Kingdom. Following its incursions in 2006 and 2010, BTV-1 reappeared in Sardinia in the autumn of 2012 and persisted in subsequent years, invading Corsica, Sicily, and mainland Italy [65]. The BTV-4 strain identified in Greece in 1999 was closely related to isolates recorded in the 1960s and 1970s from Cyprus and Turkey [26]. Additionally, BTV-2 entered Tunisia in 1999, spread to Algeria and Morocco in 2000, and subsequently advanced into the western and central Mediterranean islands as well as mainland Italy. Moreover, a distinct BTV-4 strain, unrelated to those circulating in the eastern Mediterranean basin, appears to have spread from North Africa to Spain, Portugal, and Corsica between 2003 and 2005 [25]. During 2006–2009, incursions of BTV-6, -8, and -11 were reported in Europe. The BTV-8 strain was confirmed to have originated from sub-Saharan Africa, with climatic changes and altered wind patterns suggested as contributing factors influencing vector distribution [66]. At the same time, genetically similar strains such as BTV-6 and BTV-11 were detected in Germany, the Netherlands, and Belgium. These viruses showed close genetic identity to live attenuated vaccine strains, suggesting a possible link to their unauthorized or accidental use [25].

In Israel, a different pattern of BTV spread than that observed in Europe was evident. Phylogenetic, pairwise, and BLASTn analyses of Israeli BTV-1 strains (ISR-2050/19, identified in 2019, and ISR-3279/1/21, detected in 2021) showed that they do not cluster together, indicating distinct evolutionary origins. Notably, the Israeli BTV-3 strains are most closely related to the Tunisian BTV-3 strain TUN2016/Zarsis, whereas two different European BTV-3 strains show a closer relationship with other African BTV-3 isolates. Since 2013, several distinct incursions of different BTV-3 genotypes have been recorded in Israel: one in 2013 (ISR-2019/13) and two genetically distinct genotypes in 2016 (ISR-2153/16 and ISR-2262/2/16) [53]. The strain ISR-2153/16 was likely the ancestor of the local BTV-3 strains circulating between 2016 and 2020. In 2020, a new introduction of BTV-3 occurred, and two groups of strains were detected simultaneously. Comparison of strains circulating during 2016–2020 and 2020–2023 revealed that the outer genes (Seg-2 and Seg-5) and only three of the seven internal genes (Seg-7, -8, and -10) shared a common ancestor. Since 2021, only one genotype—the most recent—of BTV-3 has been detected in Israel. Considering reassortment events, during 2016–2023, only a single case of reassortment between a BTV-3 strain and other local strains was identified among fully sequenced isolates (strain ISR-1434/1/23) [53]. Nevertheless, the recently identified and analyzed European and Israeli BTV-3 strains showed a close relationship of their Seg-7 sequences, with a minimum nt identity of 96.37%. Analysis of European BTV-3 revealed that Mediterranean BTV-3 strains are more closely related to continental European BTV-3 strains and to the Israeli BTV-6 strain ISR-2095/3/17. Notably, the newly emerging Mediterranean BTV-3, -4, and -8 strains exhibit multiple reassortment events both among themselves and with different local BTV strains.

Analyses of European BTV-8 revealed the presence of two distinct genotypes: one circulating in continental Europe since 2006, which was also introduced into Israel, and a second genotype that emerged in Mediterranean European countries in 2023. The European BTV-8 strains are more closely related to each other than to the recently emerged Israeli BTV-8 strains (2019–2023). These findings suggest a common origin for the European strains and indicate separate routes of introduction for the new BTV-8 strains into Israel, the European Mediterranean region, and continental Europe.

Detailed analysis of recently identified BTV-12 strains in Israel and the Netherlands revealed close genetic identity with the BTV-12 strain recently detected on Mayotte Island in 2024 for the majority of genes (7/10 for Israeli strains and 8/10 for the Netherlands strain). Notably, phylogenetic analysis of Seg-7 showed that all “western” BTV-12 strains clustered together, further supporting a probable common origin.

For an extended period, local Israeli BTV-4 strains remained closely related to other regional BTV-4 strains, although frequent reassortment events with multiple serotypes and genotypes were continuously observed (GeneBank, NCBI [45]). In 2023, a new BTV-4 strain was introduced into Israel and rapidly spread throughout the country. Phylogenetic and BLASTn analyses of this strain (ISR-1621/23) suggest that it has likely undergone reassortment with local Israeli strains and shares common ancestry with recently identified Israeli BTV-1, -8, and -11 strains detected between 2019 and 2023.

Notably, the most recent European BTV-4 strains cluster with the newly emerging Israeli BTV-5 based on internal gene analyses, suggesting possible co-circulation and a likely African origin for these viruses. Regarding European BTV-4, at least three distinct genotypes are currently circulating, primarily in Portugal, Spain, and France. Reassortment events between European BTV-3, -4, and -8 have also been observed, facilitating the spread of some reassorted strains into new regions, including the Mediterranean basin (e.g., BTV-3 and BTV-8).

Repeated introductions of orbiviruses into new regions in recent years may reflect changing environmental or climatic conditions influencing arbovirus transmission dynamics. Potential routes of introduction include windborne or vehicle-assisted dispersal of infected vectors, natural expansion of arthropod populations into previously uncolonized areas, and legal or illegal movement of infected domestic or wild ruminants or biological materials. According to recent studies, the majority of BTV incursions into Italy over the past 20 years have originated from Northern Africa, likely due to wind-blown dissemination of infected midges [33]. The spread of BTV-8 in Europe in 2006–2007 and of BTV-3 in the Netherlands in 2023 was probably associated with the movement of infected animals and/or midges [67].

In summary, the available data suggest that the primary origin of newly introduced arboviruses in Israel is likely Africa, although some internal genes share ancestry with viruses isolated in the Arabian Peninsula, Europe, and the Comoros Archipelago in the southwestern Indian Ocean. Many knowledge gaps remain in the molecular investigation of both historical and contemporary BTV distribution. Whole-genome sequencing of new and archived BTV strains from diverse regions is needed to clarify and complete the understanding of BTV evolution and spread.

## 5. Conclusions

All genetic analyses indicate that the recently identified Israeli and European BTV strains share common ancestors, predominantly of African origin. Notably, some genomic segments derived from the Comoros/Mayotte region or the Arabian Peninsula are more frequently observed in Israeli strains than in European BTV strains. Differences of at least 2–3% in nucleotide sequences across all genes suggest that the Israeli and European BTV strains have evolved independently over several years. These genomic distinctions indicate that there has been no direct transfer of BTV strains between Israel and Europe in the past decade.

## Figures and Tables

**Table 1:** Samples tested by group- (pan) or serotype-specific RT-qPCR.

Year	Pan-BTV Pos/Tested	Serotype Specific Pos/Tested				
		BTV-1	BTV-3	BTV-4	BTV-8	BTV-12
2021	629/1899	NT	37/192	16/193	NT	31/37
2022	221/873	71/181	53/181	50/182	NT	13/88
2023	360/1868	16/59	80/310	124/306	1/210	NT
total	1210/4640	87/240	170/683	190/681	1/210	44/125

Pos—positive, NT—not tested.

**Table 2:** Virus isolation of different BTV serotypes during 2021–2023.

Year	Serotype								Total
	BTV-1	BTV-3	BTV-4	BTV-5	BTV-6	BTV-8	BTV-11	BTV-12	
2021	2	11	3	0	0	0	4	9	29
2022	13	8	4	0	0	0	0	0	25
2023	0	2	4	5	3	1	0	0	15
total	15	21	11	5	3	1	4	9	69

**Table 3:** Information about selected Israeli BTV strains completely sequenced in the study.

Serotype	Year	Strain	Institute	Acc. Numbers
BTV-1	2021	ISR-3279/1/21	KVI	PV472031-PV472040
BTV-3	2019	ISR-2036/19	FLI	PV564881-PV564890
BTV-3	2019	ISR-2136/3/19	FLI	PV564891-PV564900
BTV-3	2020	ISR-181/1/20	FLI	PV575818, PV575821, PV585824, PV575827, PV575830, PV575833, PV575836, PV575839, PV575842, PV575845
BTV-3	2020	ISR-2658/20	FLI	PV575819, PV575822, PV585825, PV575828, PV575831, PV575834, PV575837, PV575840, PV575843, PV575846
BTV-3	2021	ISR-3126/21	FLI	PV575820, PV575823, PV585826, PV575829, PV575832, PV575835, PV575838, PV575841, PV575844, PV575847
BTV-3	2021	ISR-3301/2/21	KVI	PV553539-PV553548
BTV-3	2021	ISR-3489/21	KVI	PV553549-PV553558
BTV-3	2022	ISR-1563/22	KVI	PV495244-PV495253
BTV-3	2022	ISR-1613/22	KVI	PV524079-PV524088
BTV-3	2023	ISR-1434/1/23	KVI	PV524089-PV524094, PV535771-PV535774
BTV-3	2023	ISR-1693/1/23	KVI	PV535775-PV535777, PV553532-PV553538
BTV-4	2023	ISR-1621/23	KVI	PV582473-PV582484
BTV-5	2023	ISR-2089/7/23	KVI	PV485362-PV485371
BTV-8	2023	ISR-2178/23	KVI	PV495234-PV495243
BTV-11	2021	ISR-3265/2/21	KVI	PV485352-PV485361

Acc. numbers—accession number in NCBI website (GeneBank) [45].

## Data Availability

All data are presented in the text and in the Supplementary Materials.

## Supplementary Materials

The following supporting information can be downloaded at: https://www.mdpi.com/article/10.3390/pathogens15010038/s1, Figure S1. Phylogenetic tree of segment 2 of Israeli and global BTV-4 strains. Figure S2. Phylogenetic tree of recently emerging Israeli and global strains. Table S1. List of serotype-specific probe/primers used for identification and sequencing of bluetongue serotypes 1 and 12. Table S2. Information on closest identity of analyzed during the study recently identified Israeli bluetongue virus strains.
