# Supplementary material for: No Evidence of Direct Transmission of Emerging Bluetongue Virus Strains Between Israel and Europe Based on Genomic Analyses (2013–2023)
Source: Pathogens. 2025 Dec 28;15(1):38. doi: 10.3390/pathogens15010038 (PMC12845124; doi:10.3390/pathogens15010038)
Supplement: Supplementary file 1 [file pathogens-15-00038-s001.zip › pathogens-4031213-supplementary.pdf]

Table S1. Serotype-specific primers and probes used for the identification and sequencing of bluetongue virus serotypes 1 and 12.

serotype	name	Sequence of Oligo 5'–3'	product size (bp)	TM (°C)	source
1	1VP2-1341F	CAAGGGAACCCATGTGATT	694	53.7	this study
	1VP2-2012R	CTTGAGAGATATCCACAATGCTC			
12	BTV12-probe	FAM-CTCCACCATATGCGCCAACGATA-BHQ1	137	60	this study
	BTV12-999Fw	ATACAATTCAGGCTATCCRGA			[53]
	BTV12-1136Rw	CAATGATYGTTCCTCGTAAGC			[53]

TM- annealing temperature

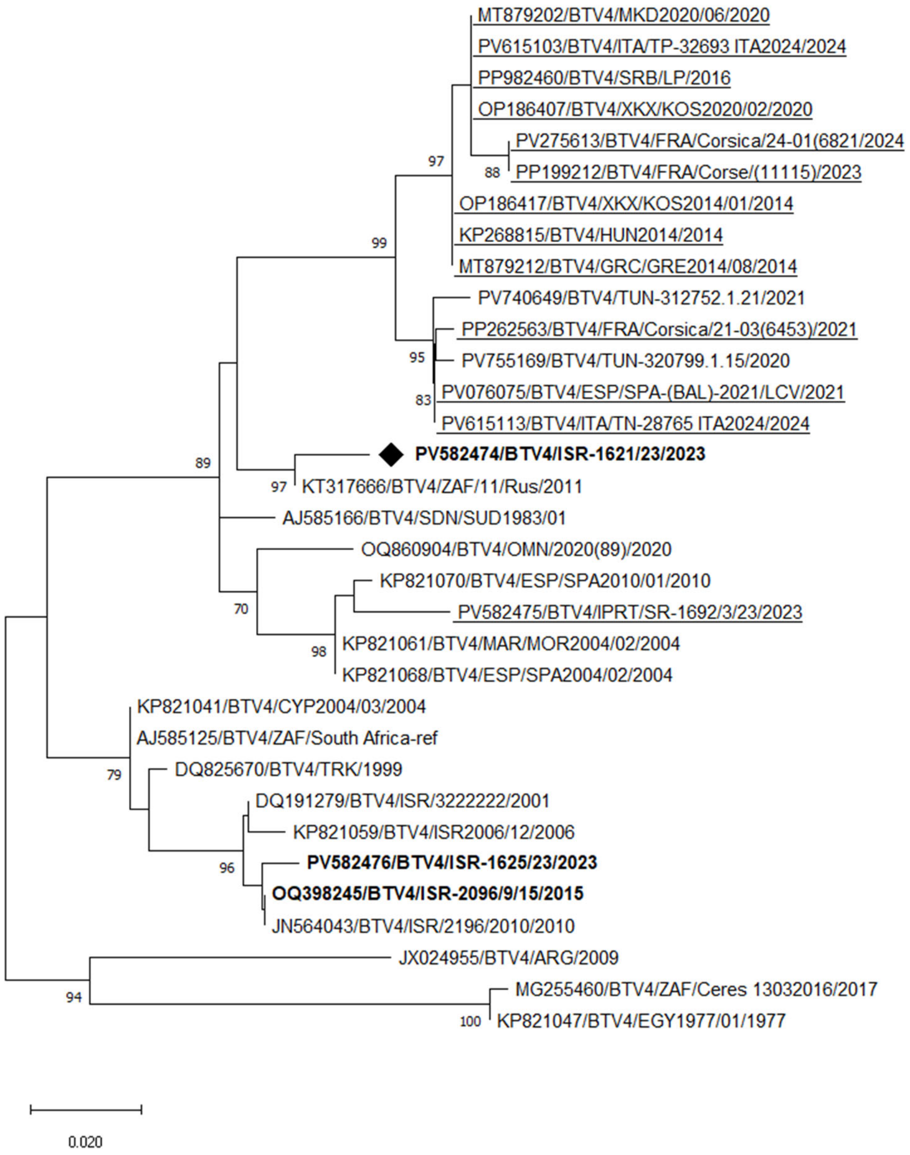

a)

Figure S1. Phylogenetic tree of segment 2 of Israeli and global BTV-4strains. Recently identified European BTV-4 (2014-2024) are underlined. Enzootic Israeli BTV-4 are marked in bold. Emerging Israeli BTV-4 signed by the rhombus. The phylogeny was inferred using the Maximum Likelihood method and Tamura-Nei model method. Statistical support for nodes was obtained by bootstrapping (1,000 replicates); only values ≥ 70 % are shown. Scale bars indicate nucleotide substitutions per site. Viruses were identified by accession number/serotype/location/isolate/year.

Phylogenetic analysis of internal genes

- Segment-1

Phylogenetic, BLASTn, and pairwise analyses revealed that the Seg-1 sequence of the Israeli BTV-1 strain ISR-2050/1/19 clustered with the BTV-8 strain from Oman (2020), sharing 97.75% nucleotide (nt) identity. Notably, both the Israeli BTV-1 ISR-2050/1/19 and the Omani BTV-8 strain clustered with BTV-3 strains that emerged in continental Europe during 2023–2024, sharing 97.34–97.37% and 97.64–97.69% nt identity, respectively. In contrast, the Israeli BTV-1 strain ISR-3279/1/21 showed a close relationship with Mediterranean BTV-1 strains circulating in 2006–2007, sharing 98.42–98.44% nt identity (Figure S2a).

Phylogenetic analysis revealed that Israeli BTV-3 strains grouped into at least two major clusters. The first, a monophyletic cluster, comprised strains isolated in 2013 and 2016 (ISR-2262/2/16) together with most strains detected between 2020 and 2023. Within this cluster, BTV-3 strains ISR-2019/13 and ISR-2262/2/16 formed a distinct subcluster that also included the BTV-11 strain ISR-3279/1/21, sharing 99.33–99.59% nt identity. Notably, this "BTV-3" cluster also contained the BTV-8 ISR-2178/23 strain, which was most closely related to BTV-3 strains from 2020–2021 (99.08% nt identity). Strains isolated during 2022–2023 formed a separate subcluster within this group, sharing 99.67–99.71% nt identity. The second major cluster comprised BTV-3 strains circulating in Israel between 2016 and 2020. This group also included the BTV-9 strain ISR-1763/3/19, which showed the highest relatedness to ISR-2153/16 and ISR-2039/19 strains (99.49% and 99.59% nt identity, respectively). Phylogenetically, strains belonging to this cluster were most closely related to South African BTV-3 strains from 2016–2017, sharing 96.34–96.80% nt identity (Figure S2a).

According to phylogenetic and pairwise analyses of sequenced Israeli BTV-4 strains, the BTV-4 identified in 2015 (strain ISR-2096/9/15) forms a distinct monophyletic branch, showing no close genetic relationship with any other known BTV strain (Figure S2a). In contrast, the recently identified strain ISR-1621/23 clustered with several historical Israeli isolates, including BTV-15 strain ISR-2006/11 (2006), BTV-24 strain ISR-2008/02 (2008), and BTV-8 strain ISR-1194/1/19 (2019) (Figure S2a), sharing 98.55%, 98.42%, and 98.51% nt identity, respectively.

Comparison with European BTV-4 strains revealed that at least two distinct genetic constellations are currently circulating in Europe. The first includes closely related BTV-4 strains that initially appeared in continental Europe in 2014 and have since been repeatedly detected. The second genotype comprises strains from Mediterranean Europe, first identified in 2023. Notably, this Mediterranean BTV-4 genotype shows an exceptionally close relationship with one constellation of BTV-3 strains detected in the Mediterranean region in 2024, sharing 99.69–99.87% nt identity. Interestingly, the recently identified Israeli BTV-5 strain ISR-2089/7/23 appears to represent an "intermediate" lineage between these two European BTV-4 genotypes, according to phylogenetic analyses. This strain shares 98.04–98.35% nt identity with the new Mediterranean BTV-3 and BTV-4 strains (2021–2024), and 98.08–98.50% with continental European BTV-4 strains.

The nt identity among the European BTV-4 strains themselves is 98.75–98.85% (Figure S2a, Table S2).

Analysis of the two European BTV-8 genotypes revealed relatively close genetic relatedness, with nt identities ranging from 97.13% to 97.51%. Moreover, the recently identified Mediterranean BTV-8 strain exhibited a remarkably high similarity (99.87% nt identity) to the Italian BTV-4 strain TN-28765 ITA2024, suggesting potential inter-serotypic reassortment events. The Israeli BTV-12 strain ISR-2717/1/20 clustered with a South African BTV-4 strain isolated in 2011, sharing 97.32% nt identity. In contrast, the European BTV-12 strain NET2024/24023518 demonstrated the closest relationship to the BTV-12 strain 24-01(3804) recently identified in Mayotte in 2024, sharing 99.04% nt identity, as supported by BLASTn and phylogenetic analyses (Figure S2a).

- Segment-3

According to phylogenetic analysis, the Israeli BTV-1 strain ISR-3279/1/21 clustered with the BTV-8 strain 5191 from Mayotte Island, detected in 2016, sharing 96.03% nt identity. BLASTn analysis, however, revealed its closest similarity to European BTV-4 strains that have circulated since 2014, particularly BTV-4 strains GRE2014/08 and KOS2014/01, with 97.49% nt identity. In contrast, the Israeli BTV-1 strain ISR-2050/19 showed the highest relatedness (98.05% nt identity) to the South African BTV-5 strain 2011/Benoni_01012015 and clustered with BTV-3 strains circulating in continental Europe since 2023, as well as with BTV-8 strains recently identified in the Mediterranean region in 2024.

Comparative analysis of these recently identified European BTV-3 and BTV-8 strains (including the reassortant Italian BTV-4 strain TN-28765 ITA2024) demonstrated 97.98–98.41% nt identity with the South African BTV-5 strain 2011/Benoni_01012015. The nucleotide identity between subclusters of BTV-3 and BTV-8 (BTV-4) was 98.59–98.67%. Furthermore, the Israeli BTV-1 strain ISR-2050/19 shared 97.50–97.79% nt identity with these European BTV-3 and BTV-8 strains (Figure S2b).

BTV-3 strains that circulated in Israel between 2016 and 2020 form a monophyletic cluster and group together with the Israeli BTV-1 strain ISR-3279/1/21, the BTV-8 strain identified in the Mediterranean region in 2024, the BTV-3 strains from continental Europe, and the South African BTV-5 strain 2011/Benoni_01012015, sharing 98.34–98.44% nt identity. In contrast, their identity with the Tunisian BTV-3 strain TUN2016/Zarzis is lower, at approximately 97.22–97.31%. The second group of Israeli BTV-3 comprised strains from 2013 (ISR-2019/13), 2016 (ISR-2262/2/16), and 2020–2023, sharing 99.31–99.78% nt identity, indicating a common ancestor. This group clustered with the recently emerged Israeli BTV-8 strain ISR-2178/23. However, accurate determination of identity is challenging due to a probable mixed Seg-3 sequence in BTV-8 ISR-2178/23, which exhibits two nt types at multiple positions. BLASTn analysis revealed that this second group of Israeli BTV-3 strains is most closely related to several South African strains belonging to BTV-2, -3, and -16, isolated in 2016–2017, sharing 97.44–97.61% nt identity (Figure S2b).

The local BTV-4 strain ISR-2096/9/15, isolated in 2015, clustered only with the local BTV-16 strain ISR-1739/6/14 from 2014, sharing 97.74% nt identity. In contrast, the recently emerged BTV-4 strain ISR-1621/23 clustered with an untyped South African strain 57/08 and several Israeli strains belonging to BTV-15, -5, and -8, suggesting the presence of this type of Seg-3 sequence in the region since at least 2006, with nt identity ranging from 97.42 to 97.99% (Figure S2b).

The emerging Israeli BTV-5 strain clustered with the South African BTV-16 strain Bethal_VR08 (2017), the South African BTV-3 strain Waterval_Boven_VR22 (2016), and the Israeli BTV-24 strain ISR2009/02. BLAST analysis further revealed 98.27% nt identity with South African BTV-3 strains Wesselsbron_VR11_2017 and Smithfield_VR33_2017 (Figure S2b).

Taking into account the BTV-11 outbreak in 2021 (representative strain ISR-3265/2/21), phylogenetic analysis revealed its closest relationship with recently identified BTV-12 strains from the Netherlands (NET2024/24023518), Mayotte (24/01 (3804)), and Israel (ISR-2717/1/20). BLASTn analysis indicated the highest nt identity of BTV-11 with the Israeli BTV-3 strain ISR-2262/2/16, sharing 99.53% nt identity (Figure S2b). Additionally, BLASTn analysis showed a very high nt identity (99.24%) between the BTV-12 strains from the Netherlands and Mayotte, while the Israeli strain ISR-2717/1/20 shared 98.90% nt identity with the other recently identified BTV-12 strains, corroborating the phylogenetic results and suggesting a probable common origin.

Regarding the recently emerging BTV-4 and BTV-3 strains circulating in the Mediterranean region of Europe, these formed a distinct subcluster with 99.75–99.89% nt identity between strains. They clustered with previously circulating BTV-1 strains from the region, likely originating from these earlier viruses, as further supported by BLASTn analysis showing 98.59–98.99% nt identity. The clustering pattern and high nt similarity with BTV-1 suggest a probable local origin of the Seg-1 genes in the Mediterranean BTV-3 and BTV-4 strains (Figure S2b).

- Segment-4

According to phylogenetic analysis, the Israeli BTV-1 strain ISR-3279/1/21 clustered with the BTV-8 strain ISR-2178/23, sharing 99.59% nt identity, while BLASTn analysis showed the closest identity (98.93%) with BTV-4 strains HUN2014 and KOS2014/01. According to updated data, the Israeli BTV-1 strain ISR-2050/1/19 clustered with the most recent Israeli BTV-3 strains (2020–2023), sharing 99.08–99.38% nt identity, while BLASTn analysis showed 98.53% nt identity of the Israeli BTV-1 strain ISR-2050/1/19 with the Tunisian BTV-3 strain TUN2016/Zarzis. At the same time, these BTV-3 strains are very closely related to each other, sharing 99.49–99.94% nt identity (Figure S2c).

Opposite to the results of phylogenetic analyses of Seg-1 and Seg-3, the Seg-4 of the BTV-3 strain ISR-1434/1/23 appears reassorted, clustering with the Israeli BTV-12 strain ISR-2717/1/20 and sharing 99.64% nt identity. These strains also clustered with the BTV-12 strain from Mayotte (24-01 (3804)) identified in 2024, which shares 98.08% and 98.92% nt identity with the BTV-3 strain ISR-1434/1/23 and the BTV-12 strain ISR-2717/1/20, respectively, correlating with BLASTn analysis. It is noteworthy that the reassorted Israeli BTV-3 and all the above-mentioned BTV-12 strains are closely related to the Israeli BTV-3 strains that circulated between 2016 and 2020, as well as to the BTV-12 strain recently detected in the Netherlands and in the UK (strain NET2024/240235180 and UKG2025/01) and the BTV-8 identified in Oman in 2020, which is also consistent with phylogenetic analysis (nt identity of 97.24–97.75%). The Israeli BTV-11 strain ISR-3265/2/21 clusters with Israeli BTV-24 strains (ISR2008/02 and ISR2009/02), and the Israeli BTV-3 strain ISR-2262/2/16 clusters with recently identified strains, sharing 98.83–98.93% and 98.72% nt identity, respectively, which also correlates with phylogenetic analysis (Figure S2c).

The local BTV-4 strain ISR-2096/9/15 clustered with the Israeli BTV-8 strain ISR-1194/1/19, forming a monophyletic group and sharing 99.34% nt identity. Interestingly, the recently identified Israeli BTV-4 strain ISR-1621/23 clustered with the Israeli BTV-3 strain ISR-2019/13, the BTV-3 strain from Tunisia (TUN2016/Zarzis), and BTV-3 strains from continental Europe, sharing 98.71–98.93% nt identity. The recently identified Israeli BTV-5 strain ISR-2089/7/23 formed a monophyletic branch, while BLASTn analysis showed its closest identity with the Tunisian BTV-2 strain TUN2000/01, sharing 98.17% nt identity (Figure S2c, Table S2). Notably, the recently emerging BTV-3 and BTV-4 strains circulating in Mediterranean European countries (2021–2024) clustered into a monophyletic group, sharing 99.34–99.90% nt identity. At the same time, phylogenetic analysis of the recently identified BTV-8 strains in Mediterranean Europe (2023–2024) revealed the formation of a monophyletic group (nt identity between strains 99.90–100%), while BLASTn analysis showed their closest identity with the Tunisian BTV-2 strain TUN2000/01, with 98.59% nt identity (Figure S2c).

- Segment-5

Similar to the results of Seg-4, phylogenetic, pairwise and BLASTn analyses revealed that Israeli BTV-1 strain ISR-2050/19 clustered with the most recent Israeli BTV-3 (2020-2023), sharing 99.37-99.77% of nt identity. Additionally, mentioned above Israeli BTV-1 and BTV-3 strains grouped with continental European BTV-3 strains, having 98.24-98.61% of nt identity (Figure S2d). Considering Israeli BTV-1 strain ISR-3279/1/21, it forms a monophyletic group with emerged between 2021 and 2023 Israeli BTV-4, -8, and -11, sharing 99.14-99.48% of nt identity. Notably, this cluster of recently identified Israeli BTV strains is grouped with recently emerged in Mediterranean Europe BTV-8 and reassorted Italian BTV-4 strain TP-32693 ITA2024, sharing 98.40-98.74% of nt identity (Figure S2d).

All Israeli BTV-3 strains of the "old genotype" that circulated in Israel between 2013 and 2020 form a monophyletic group, with nt identity among these strains ranging between 99.41% and 99.76%. BLASTn analysis with global strains showed their closest nt identity with BTV-1 strains from the Mediterranean Basin — FRA2007/18 (France) and MOR2006/06 (Morocco) — showing 97.27–98.07% nt identity (Figure S2d, Table S2).

Regarding the recently emerging Israeli BTV-5 strain ISR-2089/7/23, phylogenetic analysis revealed its clustering with the Israeli BTV-6 strain ISR-2095/3/17 (nt identity 95.49%), BTV-3 from Tunisia (strain TUN2016), and BTV-3 from Italy (strain SAR2018). BLAST analysis showed the closest identity with South African BTV-2 (isolate 1959) and BTV-3 strain Smithfield_VR33_2017, sharing 95.52% nt identity with both South African strains (Figure S2d).

Phylogenetic and pairwise analyses of all three recently identified BTV-12 topotypes from Israel, the Netherlands/the UK, and Mayotte Island revealed that they cluster together in a single group, with nt identity of 97.14–98.69% (). BLASTn analysis showed their closest identity with Mediterranean BTV-1 and BTV-4 strains circulating between 2006 and 2020, sharing 97.72–97.78% nt identity (Figure S2d, Table S2).

- Segment-7

In general, all Seg-7 sequences included in this phylogenetic analysis can be divided into two major clusters (Cluster 1 and Cluster 2; Figure S2e). Cluster 1a comprises recently identified Israeli and European BTV strains, including BTV-1, -4, -8, -9, and -11, which showed a minimum nucleotide identity of 97.40%. Cluster 1a also includes Mayotte BTV-1 and -8 strains (24-01 [3835] and 2016 [5191]), as well as the South African BTV-4 strain Rosendal VR01, isolated in 2017.

Notably, Cluster 1b contains all BTV-12 strains, including those from South Africa, Mayotte, the Netherlands, the UK, and Israel, which form a monophyletic group clearly distinct from the BTV strains in Cluster 1a (Figure S2e). Interestingly, the non–South African BTV-12 strains are closely related, sharing 99.21–99.38% nt identity (Figure S2e).

Considering the BTV-3 strains, all those analyzed in the present study clustered within Cluster 2, which included South African, Tunisian, Israeli, and European strains. In addition to the BTV-3 strains, Cluster 2 also comprises the Israeli BTV-5 (2023) and BTV-6 (2017) strains. Pairwise and BLASTn analyses revealed that the minimum nt identity among all analyzed BTV-3 strains was 96.37%, indicating a common ancestor for these viruses. However, the BTV-3 strains were further subdivided into subclusters. All Israeli BTV-3 strains grouped together, sharing 99.05–99.65% nt identity. European strains formed two distinct subclusters: one comprising strains from Mediterranean European countries and another containing strains from continental Europe, sharing up to 100% nt identity (Figure S2e).

- Segment-8

All analyzed Seg-8 sequences were divided into three clusters. Similar to Seg-7, most BTV-3 strains belonged to Cluster 1, except for the reassorted Israeli strain ISR-1434/23, the Italian strain SAR2018, and Tunisian BTV-3 strains (2016–2022). All Israeli BTV-3 strains clustered together and subdivided into two subclusters according to their genomic constellations: one comprised strains that circulated in Israel during 2016–2020, which likely originated from or shared a common ancestor with strain ISR-2153/16; the other included strains circulating between 2020 and 2023. The BTV-11 strain ISR-3265/1/21 likely acquired this gene from BTV-3, sharing 99.35% nt identity with the closely related BTV-3 strain ISR-2153/16. Notably, the recently emerged BTV-4 strain ISR-1621/23 also clustered with the main group of Israeli BTV-3 strains, showing the highest similarity to ISR-2153/16 (98.06% nt identity), as confirmed by BLASTn analysis. Two subclusters of European BTV-3 strains were also observed: strains from continental Europe clustered with the South African BTV-2 strain Queenstown_VR18_2017, sharing 97.72–97.87% nt identity. Regarding BTV-3 strains that recently emerged in the Mediterranean region of Europe, this group of viruses clustered with BTV-4 strains that appeared in the same region in 2021. This relationship was also confirmed by BLASTn analysis, which showed 99.64–99.91% nt identity between BTV-3 and BTV-4 strains, indicating a probable common origin. According to the phylogenetic analysis, they also clustered with the recently emerged Israeli BTV-5 strain ISR-2089/7/23, sharing 98.19–98.65% nt identity, and were more distantly related to a separate cluster of circulating continental European BTV-4 strains (97.87–98.40% nt identity). However, BLASTn analysis did not fully confirm this closest relationship, instead showing a higher nt identity (98.58%) with the South African BTV-5 strain Benoni_01012015 and the Tunisian BTV-2 strain TUN2000/01 (Figure S2f). Notably, BTV-4 and BTV-8 strains from the Mediterranean Basin (showing 99.82–100% identity between each other) also displayed the highest BLASTn identity with the same South African BTV-5 Benoni_01012015 and Tunisian BTV-2 TUN2000/01 strains, sharing 98.49% and 99.31% nt identity, respectively (Figure S2f).

Several BTV-3 strains belong to cluster 2. The Israeli reassorted strain ISR-1434/1/23 most likely has a local origin of this gene. According to phylogenetic analysis, strain ISR-1434/1/23 clusters with Israeli BTV-4 ISR-2096/9/15 and BTV-9 ISR-1763/3/19, sharing 99.37–99.64% nt identity. Since 2016, Tunisian strains closely related to the Italian strain SAR2018 have been circulating in the region and also belong to cluster 2. However, these Mediterranean BTV-3 strains cluster with the South African BTV-5 strain Queenstown_VR45_2017 (Figure S2f).

Updated BLASTn and phylogenetic analyses of the Israeli BTV-1 strain ISR-2050/19 revealed its clustering with the BTV-1 strain from Oman (detected in 2020), sharing 97.45% nt identity, and with the recently emerged Israeli BTV-8 strain ISR-2178/23, sharing 98.45% nt identity (Figure S2f). The Israeli BTV-1 strain ISR-3279/1/21, identified in 2021, clustered with the South African BTV-4 strain Bloemfontein_VR31_2017 (2017), sharing 98.18% nt identity, according to both phylogenetic and BLASTn analyses (Figure S2f).

The third cluster comprises BTV-12 and BTV-22 strains. It is evident that only a few BTV serotypes form this cluster. Notably, all BTV-12 strains cluster together regardless of their geographic origin, including South Africa, Mayotte Island, the Netherlands, and Israel. The Israeli BTV-12 strain ISR-2717/1/20 is most closely related to the strain from Mayotte, sharing 98.66% nt identity (Figure S2f).

- Segment-9

According to phylogenetic analysis, the recently emerged Israeli BTV-1 strain ISR-3279/1/21 clustered with several recently identified Israeli strains/serotypes, including BTV-3 (strains circulating in 2013 and from 2016 to 2020), the reassorted strain ISR-1434/1/23, two distinct genotypes of BTV-8 (ISR-1194/1/19, detected in 2019, and ISR-2178/23), and the emerging Israeli BTV-11 strain ISR-3265/2/21. As observed with other viral genes, the BTV-3 strains circulating between 2016 and 2020 formed a subcluster, sharing 99.62–99.71% nt identity, while the reassorted

strain ISR-1434/1/23 shared 97.98–98.36% nt identity with them. Since these strains also clustered with BTV-4 detected in 2010 (strain ISR-3027/3/2010) and BTV-24 in 2009 (ISR2009/02), this type of Seg-9 appears to have been circulating among local strains at least since 2009. Notably, BTV-3 strains detected in Israel during 2020–2023 are closely related to the Israeli BTV-9 strain ISR-2095/3/19 and to the Italian BTV-3 strain identified in 2018, sharing 98.82–99.41% and 98.55–98.93% nt identity, respectively (Figure S2g). Phylogenetic analysis of European BTV-3 revealed different origin of their Seg-9 sequences. BTV-3 strains from continental Europe form a monophyletic group, while BLASTn analysis indicates their closest identity with the Spanish BTV-4 strain SPA2003/03, sharing 97.04–97.43% nt identity. BTV-3 strains identified in Mediterranean European countries clustered with several Mediterranean BTV-1 and BTV-4 strains that have recently or previously circulated in the region. According to BLASTn analysis, BTV-3 strains identified in Corsica and Italy (representative strains: 24-08 (10527) and SU21113/ITA2024; Figure S2g) showed the closest identity with the Italian BTV-1 strain SAD2013, sharing 98.74–98.95% nt identity. Recently identified BTV-4 strains from Spain and France exhibited the closest relationship with the BTV-1 strain detected in Spain in 2010, sharing 97.81–97.90% nt identity. Moreover, the recently sequenced Spanish BTV-1 strain SPA2024/06 showed the highest identity with the Moroccan BTV-1 strain, sharing 99.62% nt identity (Figure S2g).

Interestingly, the recently identified Israeli BTV-4 strain ISR-1621/23 clusters with several distinct BTV strains: the Israeli BTV-15 strain ISR2006/11, Israeli and Cypriot BTV-8 strains identified in 2015–2016, the French BTV-1 strain 07-01 from 2007, and the untyped South African strain 57/08 identified in 2008. BLASTn analysis revealed comparable nt identities with all of these strains, ranging from 97.49% to 97.68%. Considering the BTV-4 strains circulating in continental Europe since 2014, they showed the closest identity with the Israeli BTV-24 strain ISR2009/02, sharing 97.81–98.95% nt identity. Analysis of the BTV-8 strains that emerged in Mediterranean Europe in 2024 indicated that they form a monophyletic branch, which also includes the Italian BTV-4 strain TP-32693 ITA 2024. This relationship was supported by BLASTn results showing 97.59–97.68% nt identity. Regarding the BTV-12 strains, the Israeli strain ISR-2717/1/20, the strain NET2024/240235180 from the Netherlands/the UK, and the strain 24-01 (3804) from Mayotte Island clustered together, sharing 98.36% and 98.73% nt identity, respectively (Figure S2g).

- Segment-10

According to phylogenetic analysis, the recently emerged Israeli BTV-1 strain ISR-3279/1/21 clustered with the Zambian BTV-7 strain ZAM MBALA MB07, sharing 99.43% nt identity. All Israeli BTV-3 viruses, except for two "first" strains belonging to different BTV-3 genotypes [44], group together in a single cluster. Within this cluster, at least two subclusters of BTV-3 can be distinguished: strains circulating between 2016 and 2020, likely derived from the ancestral strain ISR-2153/16, including the reassorted strain ISR-1434/1/23 (99.37–99.87% nt identity), and the most recently circulating strains from 2022–2023 (99.87–100% nt identity) (Figure S2h).

Phylogenetic, pairwise, and BLASTn analyses of the recently identified BTV-4 strain ISR-1621/23 showed its close relationship with several other Israeli BTV strains: BTV-8 ISR-2189/23, BTV-24 ISR2008/02, and BTV-11 ISR-3265/2/21, sharing 99.39–99.61% nt identity (Figure S2h). BLASTn analysis of the recently identified BTV-5 ISR-2089/7/23 revealed 99.87% nt identity with the Zambian BTV-5 strain ZAM KASAMA KS08, isolated in 2016. The Israeli BTV-12 strain ISR-2717/1/20 clustered with the BTV-12 strain 24-01(3804) from Mayotte, sharing 99.36% nt identity. In contrast to several internal genes, the BTV-12 strain from the Netherlands clustered with the Israeli BTV-3 strain ISR-2262/2/16, the Zambian strain ZAM_LUNDAZI_A983, and the South African BTV-7 strain Beaufort_Wes_VR43_2017, while BLASTn analysis revealed its closest nt identity (95.13%) with the South African BTV-7 strain Beaufort_Wes_VR43_2017.

Considering the European BTV-8 strains circulating since 2006, phylogenetic analysis showed that they are most closely related to most Israeli BTV-3 strains, which was confirmed by BLASTn

analysis, revealing a nt identity of 96.88–97.08%. Phylogenetic analysis of Mediterranean European BTV-8 strains and the Italian BTV-4 strain TP-32693 ITA2024 showed their close relationship with the South African BTV-3 strain Smithfield_VR38_2017, which was confirmed by BLASTn analysis, sharing 97.69–97.81% nt identity (Figure S2h).

Recently, BTV-3 and BTV-1 strains have been identified in European Mediterranean countries, clustering with the local Israeli BTV-1 strain that has been circulating since 2006. This suggests that these European strains may have acquired this gene from local Israeli strains. Notably, BTV-3 strains from France and Italy clustered with Italian BTV-1 strains, sharing 99.38–100% nt identity. At the same time, the Spanish BTV-1 strain SPA2024/06 clustered with the Moroccan BTV-1 strain MOR2009/04, sharing 99.51% nt identity. Spanish BTV-4 (strain SPA-(BAL)-2021) and Corsican BTV-4 (strain 21-03(6453)) also clustered with Mediterranean BTV-1 strains that circulated locally between 2006 and 2011 (Figure S2h).

Regarding BTV-3 strains recently identified in continental Europe, BLASTn analysis showed their closest identity with South African BTV-18 strains, sharing 98.27–98.42% nt identity. According to the phylogenetic tree, BTV-8 and BTV-4 strains identified in 2023–2024 in the Mediterranean region of Europe formed a monophyletic group (Figure S2h). BLASTn analysis revealed their closest identity with the South African BTV-13 strain VR30_2017, sharing 97.81% nt identity.

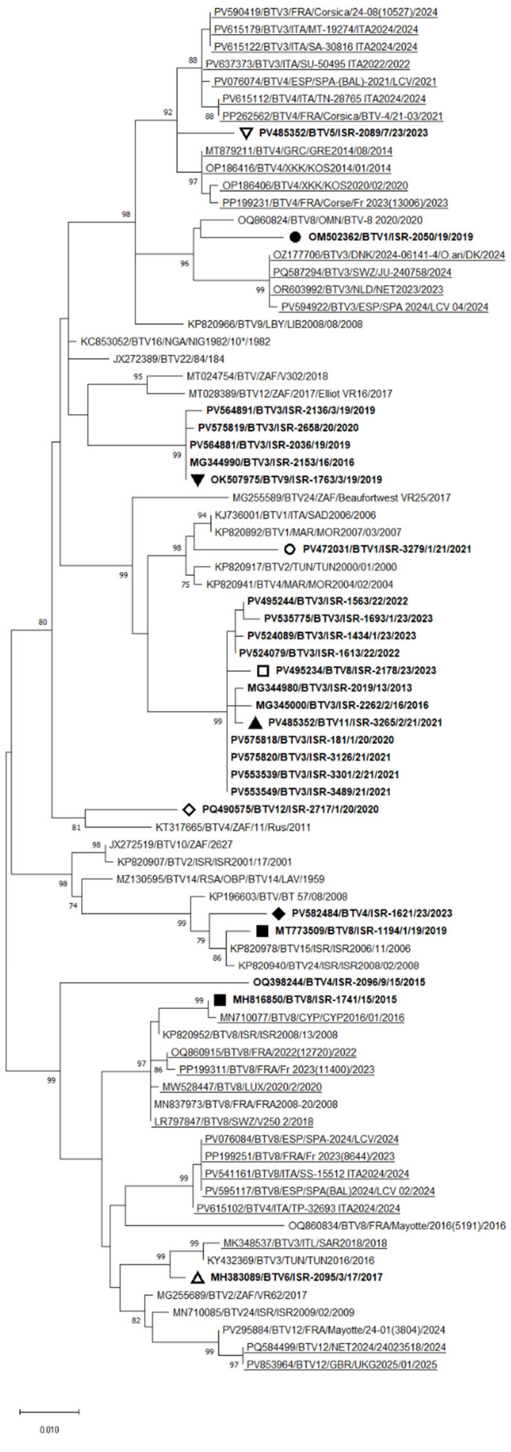
PV590419/BTV3/FRA/Corsica/24-08(10527)/2024
PV615179/BTV3/ITA/MT-19274/ITA2024/2024
PV615122/BTV3/ITA/SA-30816 ITA2024/2024
PV637373/BTV3/ITA/SU-50495 ITA2022/2022
PV076074/BTV4/ESP/SPA-(BAL)-2021/LCV/2021
PV615112/BTV4/ITA/TN-28765 ITA2024/2024
PP262562/BTV4/FRA/Corsica/BTV-4/21-03/2021
PV485352/BTV5/ISR-2089/7/23/2023
MT879211/BTV4/GRC/GRE2014/08/2014
OP186416/BTV4/XKK/KOS2014/01/2014
OP186406/BTV4/XKK/KOS2020/02/2020
PP199231/BTV4/FRA/Corse/Fr 2023(13006)/2023
OQ860824/BTV8/OMN/BTV-8 2020/2020
OM502362/BTV1/ISR-2050/19/2019
OZ177706/BTV3/DNK/2024-06141-4/O.ari/DK/2024
PQ587294/BTV3/SWZ/JU-240758/2024
OR603992/BTV3/NLD/NET2023/2023
PV594922/BTV3/ESP/SPA 2024/LCV 04/2024
KP820966/BTV9/LBY/LIB2008/08/2008
KC853052/BTV16/NGA/NIG1982/10*/1982
JX272389/BTV22/84/184
MT024754/BTV/ZAF/V302/2018
MT028389/BTV12/ZAF/2017/Elliot VR16/2017
PV564891/BTV3/ISR-2136/3/19/2019
PV575819/BTV3/ISR-2658/20/2020
PV564881/BTV3/ISR-2036/19/2019
MG344990/BTV3/ISR-2153/16/2016
OK507975/BTV9/ISR-1763/3/19/2019
MG255589/BTV24/ZAF/Beaufortwest VR25/2017
KJ736001/BTV1/ITA/SAD2006/2006
KP820892/BTV1/MAR/MOR2007/03/2007
PV472031/BTV1/ISR-3279/1/21/2021
KP820917/BTV2/TUN/TUN2000/01/2000
KP820941/BTV4/MAR/MOR2004/02/2004
PV495244/BTV3/ISR-1563/22/2022
PV535775/BTV3/ISR-1693/1/23/2023
PV524089/BTV3/ISR-1434/1/23/2023
PV524079/BTV3/ISR-1613/22/2022
PV495234/BTV8/ISR-2178/23/2023
MG344980/BTV3/ISR-2019/13/2013
MG345000/BTV3/ISR-2262/2/16/2016
PV485352/BTV11/ISR-3265/2/21/2021
PV575818/BTV3/ISR-181/1/20/2020
PV575820/BTV3/ISR-3126/21/2021
PV553539/BTV3/ISR-3301/2/21/2021
PV553549/BTV3/ISR-3489/21/2021
PQ490575/BTV12/ISR-2717/1/20/2020
KT317665/BTV4/ZAF/11/Rus/2011
JX272519/BTV10/ZAF/2627
KP820907/BTV2/ISR/ISR2001/17/2001
MZ130595/BTV14/RSA/OBP/BTV14/LAV/1959
KP196603/BTV/BT 57/08/2008
PV582484/BTV4/ISR-1621/23/2023
MT773509/BTV8/ISR-1194/1/19/2019
KP820978/BTV15/ISR/ISR2006/11/2006
KP820940/BTV24/ISR/ISR2008/02/2008
OQ398244/BTV4/ISR-2096/9/15/2015
MH816850/BTV8/ISR-1741/15/2015
MN710077/BTV8/CYP/CYP2016/01/2016
KP820952/BTV8/ISR/ISR2008/13/2008
OQ860915/BTV8/FRA/2022(12720)/2022
PP199311/BTV8/FRA/Fr 2023(11400)/2023
MW528447/BTV8/LUX/2020/2/2020
MN837973/BTV8/FRA/FRA2008-20/2008
LR797847/BTV8/SWZ/V250.2/2018
PV076084/BTV8/ESP/SPA-2024/LCV/2024
PP199251/BTV8/FRA/Fr 2023(8644)/2023
PV541161/BTV8/ITA/SS-15512 ITA2024/2024
PV595117/BTV8/ESP/SPA(BAL)2024/LCV 02/2024
PV615102/BTV4/ITA/TP-32693 ITA2024/2024
OQ860834/BTV8/FRA/Mayotte/2016(5191)/2016
MK348537/BTV3/ITL/SAR2018/2018
KY432369/BTV3/TUN/TUN2016/2016
MH383089/BTV6/ISR-2095/3/17/2017
MG255689/BTV2/ZAF/VR62/2017
MN710085/BTV24/ISR/ISR2009/02/2009
PV295884/BTV12/FRA/Mayotte/24-01(3804)/2024
PQ584499/BTV12/NET2024/24023518/2024
PV853964/BTV12/GBR/UKG2025/01/2025
0.010

a) Seg-1

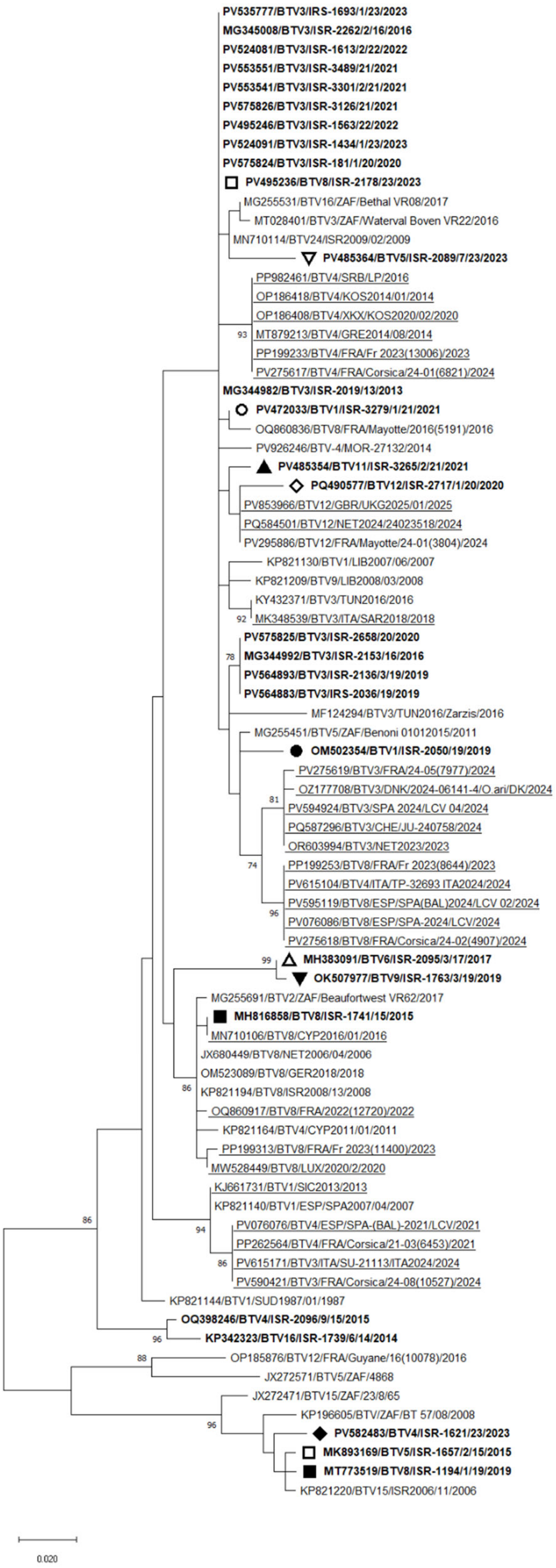

PV535777/BTV3/IRS-1693/1/23/2023
MG345008/BTV3/ISR-2262/2/16/2016
PV524081/BTV3/ISR-1613/2/22/2022
PV553551/BTV3/ISR-3489/21/2021
PV553541/BTV3/ISR-3301/2/21/2021
PV575826/BTV3/ISR-3126/21/2021
PV495246/BTV3/ISR-1563/22/2022
PV524091/BTV3/ISR-1434/1/23/2023
PV575824/BTV3/ISR-181/1/20/2020
PV495236/BTV8/ISR-2178/23/2023
MG255531/BTV16/ZAF/Bethal VR08/2017
MT028401/BTV3/ZAF/Waterval Boven VR22/2016
MN710114/BTV24/ISR2009/02/2009
PV485364/BTV5/ISR-2089/7/23/2023
PP982461/BTV4/SRB/LP/2016
OP186418/BTV4/KOS2014/01/2014
OP186408/BTV4/XKX/KOS2020/02/2020
93
MT879213/BTV4/GRE2014/08/2014
PP199233/BTV4/FRA/Fr 2023(13006)/2023
PV275617/BTV4/FRA/Corsica/24-01(6821)/2024
MG344982/BTV3/ISR-2019/13/2013
PV472033/BTV1/ISR-3279/1/21/2021
OQ860836/BTV8/FRA/Mayotte/2016(5191)/2016
PV926246/BTV-4/MOR-27132/2014
PV485354/BTV11/ISR-3265/2/21/2021
PQ490577/BTV12/ISR-2717/1/20/2020
PV853966/BTV12/GBR/UKG2025/01/2025
PQ584501/BTV12/NET2024/24023518/2024
PV295886/BTV12/FRA/Mayotte/24-01(3804)/2024
KP821130/BTV1/LIB2007/06/2007
KP821209/BTV9/LIB2008/03/2008
KY432371/BTV3/TUN2016/2016
92
MK348539/BTV3/ITA/SAR2018/2018
PV575825/BTV3/ISR-2658/20/2020
78
MG344992/BTV3/ISR-2153/16/2016
PV564893/BTV3/ISR-2136/3/19/2019
PV564883/BTV3/IRS-2036/19/2019
MF124294/BTV3/TUN2016/Zarzis/2016
MG255451/BTV5/ZAF/Benoni 01012015/2011
OM502354/BTV1/ISR-2050/19/2019
PV275619/BTV3/FRA/24-05(7977)/2024
OZ177708/BTV3/DNK/2024-06141-4/O.ari/DK/2024
81
PV594924/BTV3/SPA 2024/LCV 04/2024
PQ587296/BTV3/CHE/JU-240758/2024
OR603994/BTV3/NET2023/2023
74
PP199253/BTV8/FRA/Fr 2023(8644)/2023
PV615104/BTV4/ITA/TP-32693 ITA2024/2024
PV595119/BTV8/ESP/SPA(BAL)2024/LCV 02/2024
96
PV076086/BTV8/ESP/SPA-2024/LCV/2024
PV275618/BTV8/FRA/Corsica/24-02(4907)/2024
99
MH383091/BTV6/ISR-2095/3/17/2017
OK507977/BTV9/ISR-1763/3/19/2019
MG255691/BTV2/ZAF/Beaufortwest VR62/2017
MH816858/BTV8/ISR-1741/15/2015
MN710106/BTV8/CYP2016/01/2016
JX680449/BTV8/NET2006/04/2006
OM523089/BTV8/GER2018/2018
86
KP821194/BTV8/ISR2008/13/2008
OQ860917/BTV8/FRA/2022(12720)/2022
KP821164/BTV4/CYP2011/01/2011
PP199313/BTV8/FRA/Fr 2023(11400)/2023
MW528449/BTV8/LUX/2020/2/2020
KJ661731/BTV1/SIC2013/2013
KP821140/BTV1/ESP/SPA2007/04/2007
86
94
PV076076/BTV4/ESP/SPA-(BAL)-2021/LCV/2021
PP262564/BTV4/FRA/Corsica/21-03(6453)/2021
86
PV615171/BTV3/ITA/SU-21113/ITA2024/2024
PV590421/BTV3/FRA/Corsica/24-08(10527)/2024
KP821144/BTV1/SUD1987/01/1987
OQ398246/BTV4/ISR-2096/9/15/2015
96
KP342323/BTV16/ISR-1739/6/14/2014
88
OP185876/BTV12/FRA/Guyane/16(10078)/2016
JX272571/BTV5/ZAF/4868
JX272471/BTV15/ZAF/23/8/65
KP196605/BTV/ZAF/BT 57/08/2008
96
PV582483/BTV4/ISR-1621/23/2023
MK893169/BTV5/ISR-1657/2/15/2015
MT773519/BTV8/ISR-1194/1/19/2019
KP821220/BTV15/ISR2006/11/2006
0.020

b) Seg-3

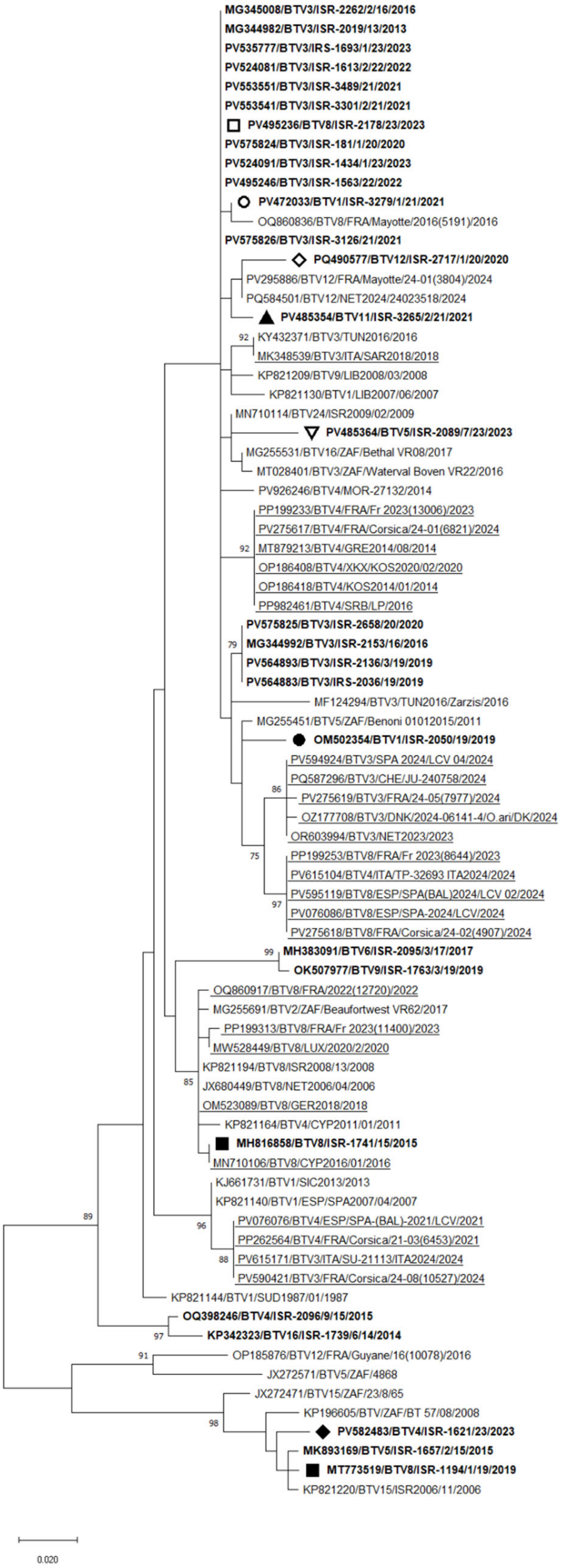

MG345008/BTV3/ISR-2262/2/16/2016
MG344982/BTV3/ISR-2019/13/2013
PV535777/BTV3/IRS-1693/1/23/2023
PV524081/BTV3/ISR-1613/2/22/2022
PV553551/BTV3/ISR-3489/21/2021
PV553541/BTV3/ISR-3301/2/21/2021
PV495236/BTV8/ISR-2178/23/2023
PV575824/BTV3/ISR-181/1/20/2020
PV524091/BTV3/ISR-1434/1/23/2023
PV495246/BTV3/ISR-1563/22/2022
PV472033/BTV1/ISR-3279/1/21/2021
OQ860836/BTV8/FRA/Mayotte/2016(5191)/2016
PV575826/BTV3/ISR-3126/21/2021
PQ490577/BTV12/ISR-2717/1/20/2020
PV295886/BTV12/FRA/Mayotte/24-01(3804)/2024
PQ584501/BTV12/NET2024/24023518/2024
PV485354/BTV11/ISR-3265/2/21/2021
KY432371/BTV3/TUN2016/2016
MK348539/BTV3/ITA/SAR2018/2018
KP821209/BTV9/LIB2008/03/2008
KP821130/BTV1/LIB2007/06/2007
MN710114/BTV24/ISR2009/02/2009
PV485364/BTV5/ISR-2089/7/23/2023
MG255531/BTV16/ZAF/Bethal VR08/2017
MT028401/BTV3/ZAF/Waterval Boven VR22/2016
PV926246/BTV4/MOR-27132/2014
PP199233/BTV4/FRA/Fr 2023(13006)/2023
PV275617/BTV4/FRA/Corsica/24-01(6821)/2024
MT879213/BTV4/GRE2014/08/2014
OP186408/BTV4/XKX/KOS2020/02/2020
OP186418/BTV4/KOS2014/01/2014
PP982461/BTV4/SRB/LP/2016
PV575825/BTV3/ISR-2658/20/2020
MG344992/BTV3/ISR-2153/16/2016
PV564893/BTV3/ISR-2136/3/19/2019
PV564883/BTV3/IRS-2036/19/2019
MF124294/BTV3/TUN2016/Zarzis/2016
MG255451/BTV5/ZAF/Benoni 01012015/2011
OM502354/BTV1/ISR-2050/19/2019
PV594924/BTV3/SPA 2024/LCV 04/2024
PQ587296/BTV3/CHE/JU-240758/2024
PV275619/BTV3/FRA/24-05(7977)/2024
OZ177708/BTV3/DNK/2024-06141-4/O.ari/DK/2024
OR603994/BTV3/NET2023/2023
PP199253/BTV8/FRA/Fr 2023(8644)/2023
PV615104/BTV4/ITA/TP-32693 ITA2024/2024
PV595119/BTV8/ESP/SPA(BAL)2024/LCV 02/2024
PV076086/BTV8/ESP/SPA-2024/LCV/2024
PV275618/BTV8/FRA/Corsica/24-02(4907)/2024
MH383091/BTV6/ISR-2095/3/17/2017
OK507977/BTV9/ISR-1763/3/19/2019
OQ860917/BTV8/FRA/2022(12720)/2022
MG255691/BTV2/ZAF/Beaufortwest VR62/2017
PP199313/BTV8/FRA/Fr 2023(11400)/2023
MW528449/BTV8/LUX/2020/2/2020
KP821194/BTV8/ISR2008/13/2008
JX680449/BTV8/NET2006/04/2006
OM523089/BTV8/GER2018/2018
KP821164/BTV4/CYP2011/01/2011
MH816858/BTV8/ISR-1741/15/2015
MN710106/BTV8/CYP2016/01/2016
KJ661731/BTV1/SIC2013/2013
KP821140/BTV1/ESP/SPA2007/04/2007
PV076076/BTV4/ESP/SPA-(BAL)-2021/LCV/2021
PP262564/BTV4/FRA/Corsica/21-03(6453)/2021
PV615171/BTV3/ITA/SU-21113/ITA2024/2024
PV590421/BTV3/FRA/Corsica/24-08(10527)/2024
KP821144/BTV1/SUD1987/01/1987
OQ398246/BTV4/ISR-2096/9/15/2015
KP342323/BTV16/ISR-1739/6/14/2014
OP185876/BTV12/FRA/Guyane/16(10078)/2016
JX272571/BTV5/ZAF/4868
JX272471/BTV15/ZAF/23/8/65
KP196605/BTV/ZAF/BT 57/08/2008
PV582483/BTV4/ISR-1621/23/2023
MK893169/BTV5/ISR-1657/2/15/2015
MT773519/BTV8/ISR-1194/1/19/2019
KP821220/BTV15/ISR2006/11/2006
92
92
79
86
75
97
99
85
89
96
88
97
91
98
0.020

c) Seg-4

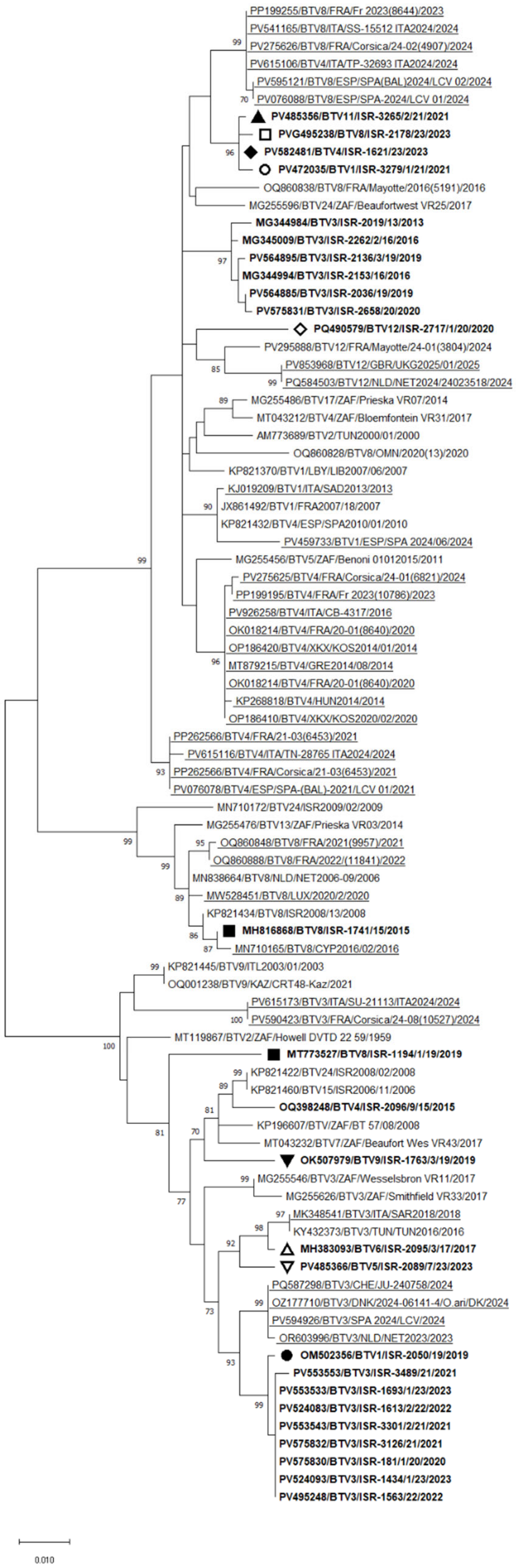
PP199255/BTV8/FRA/Fr 2023(8644)/2023
PV541165/BTV8/ITA/SS-15512 ITA2024/2024
PV275626/BTV8/FRA/Corsica/24-02(4907)/2024
PV615106/BTV4/ITA/TP-32693 ITA2024/2024
PV595121/BTV8/ESP/SPA(BAL)2024/LCV 02/2024
PV076088/BTV8/ESP/SPA-2024/LCV 01/2024
PV485356/BTV11/ISR-3265/2/21/2021
PVG495238/BTV8/ISR-2178/23/2023
PV582481/BTV4/ISR-1621/23/2023
PV472035/BTV1/ISR-3279/1/21/2021
OQ860838/BTV8/FRA/Mayotte/2016(5191)/2016
MG255596/BTV24/ZAF/Beaufortwest VR25/2017
MG344984/BTV3/ISR-2019/13/2013
MG345009/BTV3/ISR-2262/2/16/2016
PV564895/BTV3/ISR-2136/3/19/2019
MG344994/BTV3/ISR-2153/16/2016
PV564885/BTV3/ISR-2036/19/2019
PV575831/BTV3/ISR-2658/20/2020
PQ490579/BTV12/ISR-2717/1/20/2020
PV295888/BTV12/FRA/Mayotte/24-01(3804)/2024
PV853968/BTV12/GBR/UKG2025/01/2025
PQ584503/BTV12/NLD/NET2024/24023518/2024
MG255486/BTV17/ZAF/Prieska VR07/2014
MT043212/BTV4/ZAF/Bloemfontein VR31/2017
AM773689/BTV2/TUN2000/01/2000
OQ860828/BTV8/OMN/2020(13)/2020
KP821370/BTV1/LBY/LIB2007/06/2007
KJ019209/BTV1/ITA/SAD2013/2013
JX861492/BTV1/FRA2007/18/2007
KP821432/BTV4/ESP/SPA2010/01/2010
PV459733/BTV1/ESP/SPA 2024/06/2024
MG255456/BTV5/ZAF/Benoni 01012015/2011
PV275625/BTV4/FRA/Corsica/24-01(6821)/2024
PP199195/BTV4/FRA/Fr 2023(10786)/2023
PV926258/BTV4/ITA/CB-4317/2016
OK018214/BTV4/FRA/20-01(8640)/2020
OP186420/BTV4/XKX/KOS2014/01/2014
MT879215/BTV4/GRE2014/08/2014
OK018214/BTV4/FRA/20-01(8640)/2020
KP268818/BTV4/HUN2014/2014
OP186410/BTV4/XKX/KOS2020/02/2020
PP262566/BTV4/FRA/21-03(6453)/2021
PV615116/BTV4/ITA/TN-28765 ITA2024/2024
PP262566/BTV4/FRA/Corsica/21-03(6453)/2021
PV076078/BTV4/ESP/SPA-(BAL)-2021/LCV 01/2021
MN710172/BTV24/ISR2009/02/2009
MG255476/BTV13/ZAF/Prieska VR03/2014
OQ860848/BTV8/FRA/2021(9957)/2021
OQ860888/BTV8/FRA/2022/(11841)/2022
MN838664/BTV8/NLD/NET2006-09/2006
MW528451/BTV8/LUX/2020/2/2020
KP821434/BTV8/ISR2008/13/2008
MH816868/BTV8/ISR-1741/15/2015
MN710165/BTV8/CYP2016/02/2016
KP821445/BTV9/ITL2003/01/2003
OQ001238/BTV9/KAZ/CRT48-Kaz/2021
PV615173/BTV3/ITA/SU-21113/ITA2024/2024
PV590423/BTV3/FRA/Corsica/24-08(10527)/2024
MT119867/BTV2/ZAF/Howell DVTD 22 59/1959
MT773527/BTV8/ISR-1194/1/19/2019
KP821422/BTV24/ISR2008/02/2008
KP821460/BTV15/ISR2006/11/2006
OQ398248/BTV4/ISR-2096/9/15/2015
KP196607/BTV/ZAF/BT 57/08/2008
MT043232/BTV7/ZAF/Beaufort Wes VR43/2017
OK507979/BTV9/ISR-1763/3/19/2019
MG255546/BTV3/ZAF/Wesselsbron VR11/2017
MG255626/BTV3/ZAF/Smithfield VR33/2017
MK348541/BTV3/ITA/SAR2018/2018
KY432373/BTV3/TUN/TUN2016/2016
MH383093/BTV6/ISR-2095/3/17/2017
PV485366/BTV5/ISR-2089/7/23/2023
PQ587298/BTV3/CHE/JU-240758/2024
OZ177710/BTV3/DNK/2024-06141-4/O.ari/DK/2024
PV594926/BTV3/SPA 2024/LCV/2024
OR603996/BTV3/NLD/NET2023/2023
OM502356/BTV1/ISR-2050/19/2019
PV553553/BTV3/ISR-3489/21/2021
PV553533/BTV3/ISR-1693/1/23/2023
PV524083/BTV3/ISR-1613/2/22/2022
PV553543/BTV3/ISR-3301/2/21/2021
PV575832/BTV3/ISR-3126/21/2021
PV575830/BTV3/ISR-181/1/20/2020
PV524093/BTV3/ISR-1434/1/23/2023
PV495248/BTV3/ISR-1563/22/2022
0.010

d) Seg-5

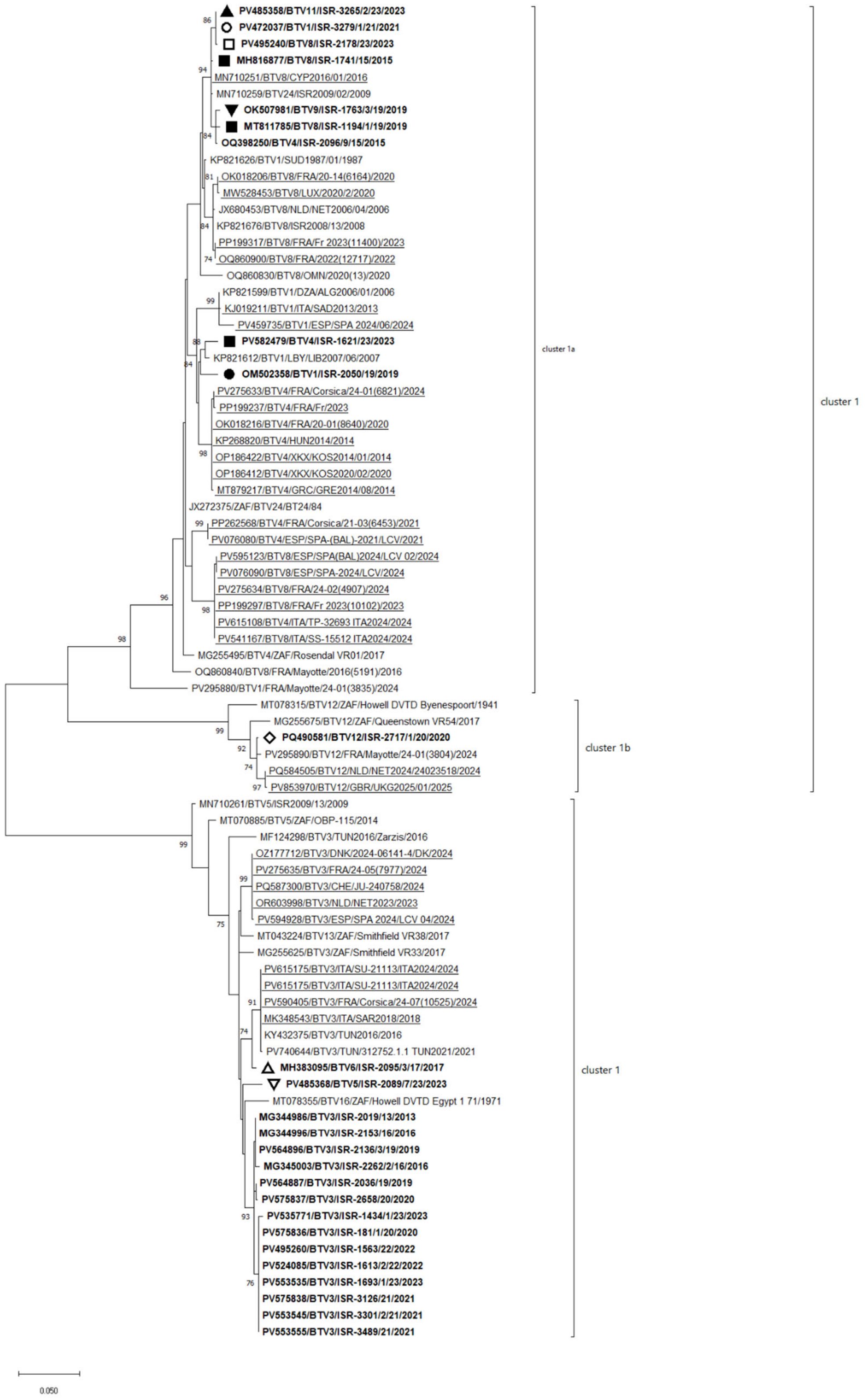
PV485358/BTV11/ISR-3265/2/23/2023
PV472037/BTV1/ISR-3279/1/21/2021
PV495240/BTV8/ISR-2178/23/2023
MH816877/BTV8/ISR-1741/15/2015
MN710251/BTV8/CYP2016/01/2016
MN710259/BTV24/ISR2009/02/2009
OK507981/BTV9/ISR-1763/3/19/2019
MT811785/BTV8/ISR-1194/1/19/2019
OQ398250/BTV4/ISR-2096/9/15/2015
KP821626/BTV1/SUD1987/01/1987
OK018206/BTV8/FRA/20-14(6164)/2020
MW528453/BTV8/LUX/2020/2/2020
JX680453/BTV8/NLD/NET2006/04/2006
KP821676/BTV8/ISR2008/13/2008
PP199317/BTV8/FRA/Fr 2023(11400)/2023
OQ860900/BTV8/FRA/2022(12717)/2022
OQ860830/BTV8/OMN/2020(13)/2020
KP821599/BTV1/DZA/ALG2006/01/2006
KJ019211/BTV1/ITA/SAD2013/2013
PV459735/BTV1/ESP/SPA 2024/06/2024
PV582479/BTV4/ISR-1621/23/2023
KP821612/BTV1/LBY/LIB2007/06/2007
OM502358/BTV1/ISR-2050/19/2019
PV275633/BTV4/FRA/Corsica/24-01(6821)/2024
PP199237/BTV4/FRA/Fr/2023
OK018216/BTV4/FRA/20-01(8640)/2020
KP268820/BTV4/HUN2014/2014
OP186422/BTV4/XKX/KOS2014/01/2014
OP186412/BTV4/XKX/KOS2020/02/2020
MT879217/BTV4/GRC/GRE2014/08/2014
JX272375/ZAF/BTV24/BT24/84
PP262568/BTV4/FRA/Corsica/21-03(6453)/2021
PV076080/BTV4/ESP/SPA-(BAL)-2021/LCV/2021
PV595123/BTV8/ESP/SPA(BAL)2024/LCV 02/2024
PV076090/BTV8/ESP/SPA-2024/LCV/2024
PV275634/BTV8/FRA/24-02(4907)/2024
PP199297/BTV8/FRA/Fr 2023(10102)/2023
PV615108/BTV4/ITA/TP-32693 ITA2024/2024
PV541167/BTV8/ITA/SS-15512 ITA2024/2024
MG255495/BTV4/ZAF/Rosendal VR01/2017
OQ860840/BTV8/FRA/Mayotte/2016(5191)/2016
PV295880/BTV1/FRA/Mayotte/24-01(3835)/2024
MT078315/BTV12/ZAF/Howell DVTD Byenespoort/1941
MG255675/BTV12/ZAF/Queenstown VR54/2017
PQ490581/BTV12/ISR-2717/1/20/2020
PV295890/BTV12/FRA/Mayotte/24-01(3804)/2024
PQ584505/BTV12/NLD/NET2024/24023518/2024
PV853970/BTV12/GBR/UKG2025/01/2025
MN710261/BTV5/ISR2009/13/2009
MT070885/BTV5/ZAF/OBP-115/2014
MF124298/BTV3/TUN2016/Zarzis/2016
OZ177712/BTV3/DNK/2024-06141-4/DK/2024
PV275635/BTV3/FRA/24-05(7977)/2024
PQ587300/BTV3/CHE/JU-240758/2024
OR603998/BTV3/NLD/NET2023/2023
PV594928/BTV3/ESP/SPA 2024/LCV 04/2024
MT043224/BTV13/ZAF/Smithfield VR38/2017
MG255625/BTV3/ZAF/Smithfield VR33/2017
PV615175/BTV3/ITA/SU-21113/ITA2024/2024
PV615175/BTV3/ITA/SU-21113/ITA2024/2024
PV590405/BTV3/FRA/Corsica/24-07(10525)/2024
MK348543/BTV3/ITA/SAR2018/2018
KY432375/BTV3/TUN2016/2016
PV740644/BTV3/TUN/312752.1.1 TUN2021/2021
MH383095/BTV6/ISR-2095/3/17/2017
PV485368/BTV5/ISR-2089/7/23/2023
MT078355/BTV16/ZAF/Howell DVTD Egypt 1 71/1971
MG344986/BTV3/ISR-2019/13/2013
MG344996/BTV3/ISR-2153/16/2016
PV564896/BTV3/ISR-2136/3/19/2019
MG345003/BTV3/ISR-2262/2/16/2016
PV564887/BTV3/ISR-2036/19/2019
PV575837/BTV3/ISR-2658/20/2020
PV535771/BTV3/ISR-1434/1/23/2023
PV575836/BTV3/ISR-181/1/20/2020
PV495260/BTV3/ISR-1563/22/2022
PV524085/BTV3/ISR-1613/2/22/2022
PV553535/BTV3/ISR-1693/1/23/2023
PV575838/BTV3/ISR-3126/21/2021
PV553545/BTV3/ISR-3301/2/21/2021
PV553555/BTV3/ISR-3489/21/2021
cluster 1a
cluster 1
cluster 1b
cluster 1
0.050

e) Seg-7

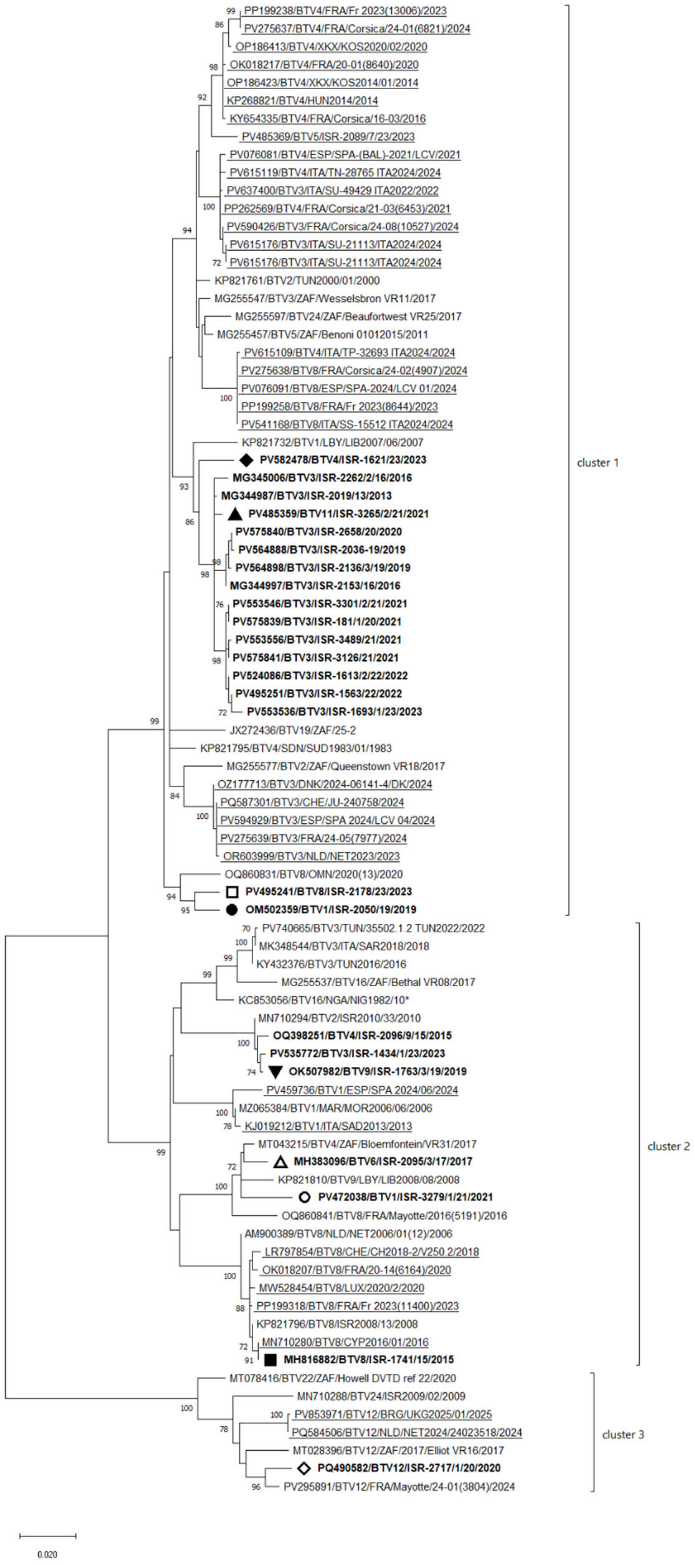

PP199238/BTV4/FRA/Fr 2023(13006)/2023
PV275637/BTV4/FRA/Corsica/24-01(6821)/2024
OP186413/BTV4/XKX/KOS2020/02/2020
OK018217/BTV4/FRA/20-01(8640)/2020
OP186423/BTV4/XKX/KOS2014/01/2014
KP268821/BTV4/HUN2014/2014
KY654335/BTV4/FRA/Corsica/16-03/2016
PV485369/BTV5/ISR-2089/7/23/2023
PV076081/BTV4/ESP/SPA-(BAL)-2021/LCV/2021
PV615119/BTV4/ITA/TN-28765 ITA2024/2024
PV637400/BTV3/ITA/SU-49429 ITA2022/2022
PP262569/BTV4/FRA/Corsica/21-03(6453)/2021
PV590426/BTV3/FRA/Corsica/24-08(10527)/2024
PV615176/BTV3/ITA/SU-21113/ITA2024/2024
PV615176/BTV3/ITA/SU-21113/ITA2024/2024
KP821761/BTV2/TUN2000/01/2000
MG255547/BTV3/ZAF/Wesselsbron VR11/2017
MG255597/BTV24/ZAF/Beaufortwest VR25/2017
MG255457/BTV5/ZAF/Benoni 01012015/2011
PV615109/BTV4/ITA/TP-32693 ITA2024/2024
PV275638/BTV8/FRA/Corsica/24-02(4907)/2024
PV076091/BTV8/ESP/SPA-2024/LCV 01/2024
PP199258/BTV8/FRA/Fr 2023(8644)/2023
PV541168/BTV8/ITA/SS-15512 ITA2024/2024
KP821732/BTV1/LBY/LIB2007/06/2007
PV582478/BTV4/ISR-1621/23/2023
MG345006/BTV3/ISR-2262/2/16/2016
MG344987/BTV3/ISR-2019/13/2013
PV485359/BTV11/ISR-3265/2/21/2021
PV575840/BTV3/ISR-2658/20/2020
PV564888/BTV3/ISR-2036-19/2019
PV564898/BTV3/ISR-2136/3/19/2019
MG344997/BTV3/ISR-2153/16/2016
PV553546/BTV3/ISR-3301/2/21/2021
PV575839/BTV3/ISR-181/1/20/2021
PV553556/BTV3/ISR-3489/21/2021
PV575841/BTV3/ISR-3126/21/2021
PV524086/BTV3/ISR-1613/2/22/2022
PV495251/BTV3/ISR-1563/22/2022
PV553536/BTV3/ISR-1693/1/23/2023
JX272436/BTV19/ZAF/25-2
KP821795/BTV4/SDN/SUD1983/01/1983
MG255577/BTV2/ZAF/Queenstown VR18/2017
OZ177713/BTV3/DNK/2024-06141-4/DK/2024
PQ587301/BTV3/CHE/JU-240758/2024
PV594929/BTV3/ESP/SPA 2024/LCV 04/2024
PV275639/BTV3/FRA/24-05(7977)/2024
OR603999/BTV3/NLD/NET2023/2023
OQ860831/BTV8/OMN/2020(13)/2020
PV495241/BTV8/ISR-2178/23/2023
OM502359/BTV1/ISR-2050/19/2019
cluster 1
PV740665/BTV3/TUN/35502.1.2 TUN2022/2022
MK348544/BTV3/ITA/SAR2018/2018
KY432376/BTV3/TUN2016/2016
MG255537/BTV16/ZAF/Bethal VR08/2017
KC853056/BTV16/NGA/NIG1982/10*
MN710294/BTV2/ISR2010/33/2010
OQ398251/BTV4/ISR-2096/9/15/2015
PV535772/BTV3/ISR-1434/1/23/2023
OK507982/BTV9/ISR-1763/3/19/2019
PV459736/BTV1/ESP/SPA 2024/06/2024
MZ065384/BTV1/MAR/MOR2006/06/2006
KJ019212/BTV1/ITA/SAD2013/2013
MT043215/BTV4/ZAF/Bloemfontein/VR31/2017
MH383096/BTV6/ISR-2095/3/17/2017
KP821810/BTV9/LBY/LIB2008/08/2008
PV472038/BTV1/ISR-3279/1/21/2021
OQ860841/BTV8/FRA/Mayotte2016(5191)/2016
AM900389/BTV8/NLD/NET2006/01(12)/2006
LR797854/BTV8/CHE/CH2018-2/V250 2/2018
OK018207/BTV8/FRA/20-14(6164)/2020
MW528454/BTV8/LUX/2020/2/2020
PP199318/BTV8/FRA/Fr 2023(11400)/2023
KP821796/BTV8/ISR2008/13/2008
MN710280/BTV8/CYP2016/01/2016
MH816882/BTV8/ISR-1741/15/2015
cluster 2
MT078416/BTV22/ZAF/Howell DVTD ref 22/2020
MN710288/BTV24/ISR2009/02/2009
PV853971/BTV12/BRG/UKG2025/01/2025
PQ584506/BTV12/NLD/NET2024/24023518/2024
MT028396/BTV12/ZAF/2017/Elliot VR16/2017
PQ490582/BTV12/ISR-2717/1/20/2020
PV295891/BTV12/FRA/Mayotte/24-01(3804)/2024
cluster 3
0.020

f) Seg-8

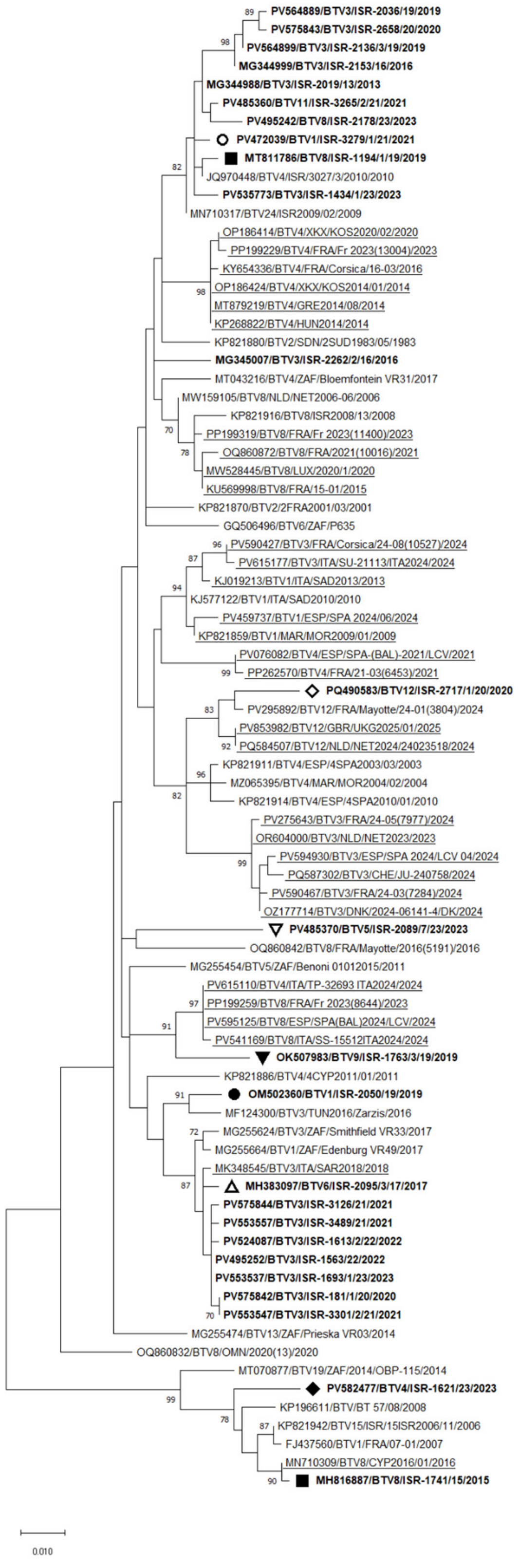
PV564889/BTV3/ISR-2036/19/2019
PV575843/BTV3/ISR-2658/20/2020
PV564899/BTV3/ISR-2136/3/19/2019
MG344999/BTV3/ISR-2153/16/2016
MG344988/BTV3/ISR-2019/13/2013
PV485360/BTV11/ISR-3265/2/21/2021
PV495242/BTV8/ISR-2178/23/2023
PV472039/BTV1/ISR-3279/1/21/2021
MT811786/BTV8/ISR-1194/1/19/2019
JQ970448/BTV4/ISR/3027/3/2010/2010
PV535773/BTV3/ISR-1434/1/23/2023
MN710317/BTV24/ISR2009/02/2009
OP186414/BTV4/XKX/KOS2020/02/2020
PP199229/BTV4/FRA/Fr 2023(13004)/2023
KY654336/BTV4/FRA/Corsica/16-03/2016
OP186424/BTV4/XKX/KOS2014/01/2014
MT879219/BTV4/GRE2014/08/2014
KP268822/BTV4/HUN2014/2014
KP821880/BTV2/SDN/2SUD1983/05/1983
MG345007/BTV3/ISR-2262/2/16/2016
MT043216/BTV4/ZAF/Bloemfontein VR31/2017
MW159105/BTV8/NLD/NET2006-06/2006
KP821916/BTV8/ISR2008/13/2008
PP199319/BTV8/FRA/Fr 2023(11400)/2023
OQ860872/BTV8/FRA/2021(10016)/2021
MW528445/BTV8/LUX/2020/1/2020
KU569998/BTV8/FRA/15-01/2015
KP821870/BTV2/2FRA2001/03/2001
GQ506496/BTV6/ZAF/P635
PV590427/BTV3/FRA/Corsica/24-08(10527)/2024
PV615177/BTV3/ITA/SU-21113/ITA2024/2024
KJ019213/BTV1/ITA/SAD2013/2013
KJ577122/BTV1/ITA/SAD2010/2010
PV459737/BTV1/ESP/SPA 2024/06/2024
KP821859/BTV1/MAR/MOR2009/01/2009
PV076082/BTV4/ESP/SPA-(BAL)-2021/LCV/2021
PP262570/BTV4/FRA/21-03(6453)/2021
PQ490583/BTV12/ISR-2717/1/20/2020
PV295892/BTV12/FRA/Mayotte/24-01(3804)/2024
PV853982/BTV12/GBR/UKG2025/01/2025
PQ584507/BTV12/NLD/NET2024/24023518/2024
KP821911/BTV4/ESP/4SPA2003/03/2003
MZ065395/BTV4/MAR/MOR2004/02/2004
KP821914/BTV4/ESP/4SPA2010/01/2010
PV275643/BTV3/FRA/24-05(7977)/2024
OR604000/BTV3/NLD/NET2023/2023
PV594930/BTV3/ESP/SPA 2024/LCV 04/2024
PQ587302/BTV3/CHE/JU-240758/2024
PV590467/BTV3/FRA/24-03(7284)/2024
OZ177714/BTV3/DNK/2024-06141-4/DK/2024
PV485370/BTV5/ISR-2089/7/23/2023
OQ860842/BTV8/FRA/Mayotte/2016(5191)/2016
MG255454/BTV5/ZAF/Benoni 01012015/2011
PV615110/BTV4/ITA/TP-32693 ITA2024/2024
PP199259/BTV8/FRA/Fr 2023(8644)/2023
PV595125/BTV8/ESP/SPA(BAL)2024/LCV/2024
PV541169/BTV8/ITA/SS-15512ITA2024/2024
OK507983/BTV9/ISR-1763/3/19/2019
KP821886/BTV4/4CYP2011/01/2011
OM502360/BTV1/ISR-2050/19/2019
MF124300/BTV3/TUN2016/Zarzis/2016
MG255624/BTV3/ZAF/Smithfield VR33/2017
MG255664/BTV1/ZAF/Edenburg VR49/2017
MK348545/BTV3/ITA/SAR2018/2018
MH383097/BTV6/ISR-2095/3/17/2017
PV575844/BTV3/ISR-3126/21/2021
PV553557/BTV3/ISR-3489/21/2021
PV524087/BTV3/ISR-1613/2/22/2022
PV495252/BTV3/ISR-1563/22/2022
PV553537/BTV3/ISR-1693/1/23/2023
PV575842/BTV3/ISR-181/1/20/2020
PV553547/BTV3/ISR-3301/2/21/2021
MG255474/BTV13/ZAF/Prieska VR03/2014
OQ860832/BTV8/OMN/2020(13)/2020
MT070877/BTV19/ZAF/2014/OBP-115/2014
PV582477/BTV4/ISR-1621/23/2023
KP196611/BTV/BT 57/08/2008
KP821942/BTV15/ISR/15ISR2006/11/2006
FJ437560/BTV1/FRA/07-01/2007
MN710309/BTV8/CYP2016/01/2016
MH816887/BTV8/ISR-1741/15/2015
0.010

g) Seg-9

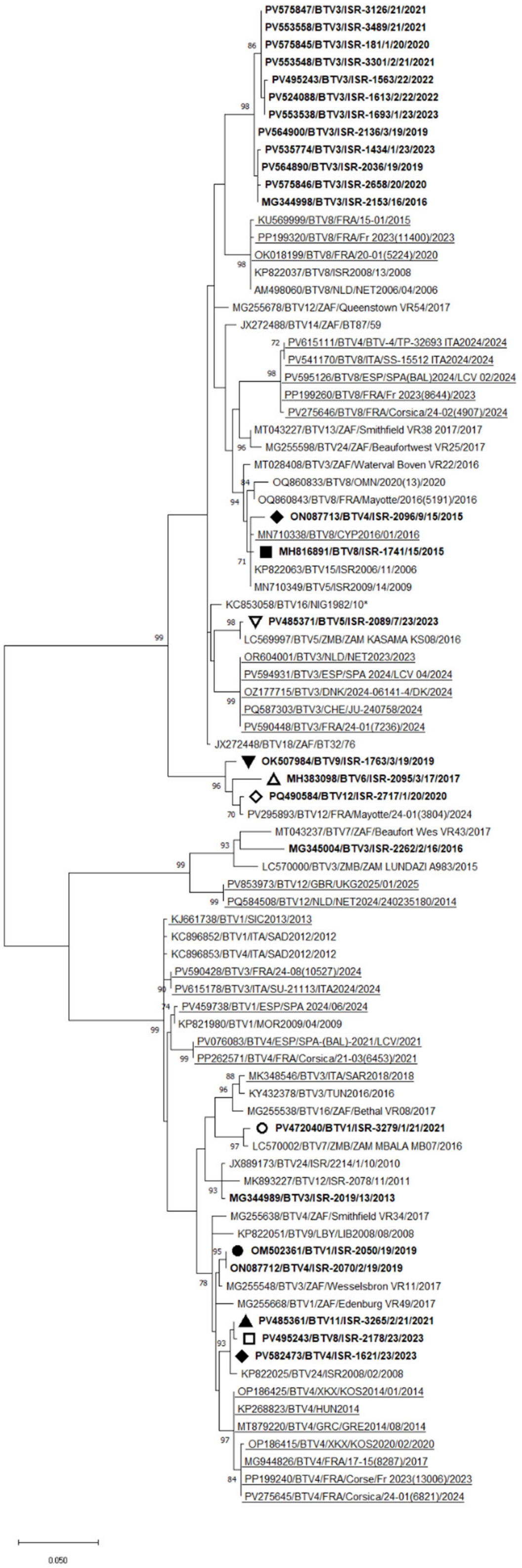
PV575847/BTV3/ISR-3126/21/2021
PV553558/BTV3/ISR-3489/21/2021
86
PV575845/BTV3/ISR-181/1/20/2020
PV553548/BTV3/ISR-3301/2/21/2021
PV495243/BTV3/ISR-1563/22/2022
PV524088/BTV3/ISR-1613/2/22/2022
98
PV553538/BTV3/ISR-1693/1/23/2023
PV564900/BTV3/ISR-2136/3/19/2019
PV535774/BTV3/ISR-1434/1/23/2023
PV564890/BTV3/ISR-2036/19/2019
PV575846/BTV3/ISR-2658/20/2020
MG344998/BTV3/ISR-2153/16/2016
KU569999/BTV8/FRA/15-01/2015
PP199320/BTV8/FRA/Fr 2023(11400)/2023
OK018199/BTV8/FRA/20-01(5224)/2020
98
KP822037/BTV8/ISR2008/13/2008
AM498060/BTV8/NLD/NET2006/04/2006
MG255678/BTV12/ZAF/Queenstown VR54/2017
JX272488/BTV14/ZAF/BT87/59
72
PV615111/BTV4/BTV-4/TP-32693 ITA2024/2024
PV541170/BTV8/ITA/SS-15512 ITA2024/2024
98
PV595126/BTV8/ESP/SPA(BAL)2024/LCV 02/2024
PP199260/BTV8/FRA/Fr 2023(8644)/2023
PV275646/BTV8/FRA/Corsica/24-02(4907)/2024
MT043227/BTV13/ZAF/Smithfield VR38 2017/2017
96
MG255598/BTV24/ZAF/Beaufortwest VR25/2017
MT028408/BTV3/ZAF/Waterval Boven VR22/2016
84
OQ860833/BTV8/OMN/2020(13)/2020
94
OQ860843/BTV8/FRA/Mayotte/2016(5191)/2016
ON087713/BTV4/ISR-2096/9/15/2015
MN710338/BTV8/CYP2016/01/2016
MH816891/BTV8/ISR-1741/15/2015
71
KP822063/BTV15/ISR2006/11/2006
MN710349/BTV5/ISR2009/14/2009
KC853058/BTV16/NIG1982/10*
99
98
PV485371/BTV5/ISR-2089/7/23/2023
LC569997/BTV5/ZMB/ZAM KASAMA KS08/2016
OR604001/BTV3/NLD/NET2023/2023
PV594931/BTV3/ESP/SPA 2024/LCV 04/2024
OZ177715/BTV3/DNK/2024-06141-4/DK/2024
99
PQ587303/BTV3/CHE/JU-240758/2024
PV590448/BTV3/FRA/24-01(7236)/2024
JX272448/BTV18/ZAF/BT32/76
OK507984/BTV9/ISR-1763/3/19/2019
96
MH383098/BTV6/ISR-2095/3/17/2017
PQ490584/BTV12/ISR-2717/1/20/2020
70
PV295893/BTV12/FRA/Mayotte/24-01(3804)/2024
MT043237/BTV7/ZAF/Beaufort Wes VR43/2017
93
MG345004/BTV3/ISR-2262/2/16/2016
99
LC570000/BTV3/ZMB/ZAM LUNDAZI A983/2015
PV853973/BTV12/GBR/UKG2025/01/2025
99
PQ584508/BTV12/NLD/NET2024/240235180/2014
KJ661738/BTV1/SIC2013/2013
KC896852/BTV1/ITA/SAD2012/2012
KC896853/BTV4/ITA/SAD2012/2012
PV590428/BTV3/FRA/24-08(10527)/2024
90
PV615178/BTV3/ITA/SU-21113/ITA2024/2024
74
PV459738/BTV1/ESP/SPA 2024/06/2024
99
KP821980/BTV1/MOR2009/04/2009
PV076083/BTV4/ESP/SPA-(BAL)-2021/LCV/2021
99
PP262571/BTV4/FRA/Corsica/21-03(6453)/2021
88
MK348546/BTV3/ITA/SAR2018/2018
96
KY432378/BTV3/TUN2016/2016
MG255538/BTV16/ZAF/Bethal VR08/2017
PV472040/BTV1/ISR-3279/1/21/2021
97
LC570002/BTV7/ZMB/ZAM MBALA MB07/2016
JX889173/BTV24/ISR/2214/1/10/2010
MK893227/BTV12/ISR-2078/11/2011
93
MG344989/BTV3/ISR-2019/13/2013
MG255638/BTV4/ZAF/Smithfield VR34/2017
KP822051/BTV9/LBY/LIB2008/08/2008
95
OM502361/BTV1/ISR-2050/19/2019
ON087712/BTV4/ISR-2070/2/19/2019
78
MG255548/BTV3/ZAF/Wesselsbron VR11/2017
MG255668/BTV1/ZAF/Edenburg VR49/2017
PV485361/BTV11/ISR-3265/2/21/2021
PV495243/BTV8/ISR-2178/23/2023
93
PV582473/BTV4/ISR-1621/23/2023
KP822025/BTV24/ISR2008/02/2008
OP186425/BTV4/XKX/KOS2014/01/2014
KP268823/BTV4/HUN2014
MT879220/BTV4/GRC/GRE2014/08/2014
97
OP186415/BTV4/XKX/KOS2020/02/2020
MG944826/BTV4/FRA/17-15(8287)/2017
84
PP199240/BTV4/FRA/Corse/Fr 2023(13006)/2023
PV275645/BTV4/FRA/Corsica/24-01(6821)/2024
0.050

h) Seg-10

Figure S2. Phylogenetic tree of recently emerging Israeli and global strains. a) segment 1; b) segment 3; c) segment 4; d) segment 5; e) segment 7; f) segment 8; g) segment 9; h) segment 10. Recently identified European BTV strains (2013-2024) are underlined. All Israeli BTV (2013-2023) are signed in bold. Israeli BTV-1 strain ISR-2050/1/19 is signed by the black circle; Israeli BTV-1 strain ISR-3279/1/21 is signed by the empty circle; Israeli BTV-4 strain ISR-1621/23 is signed by the black rhombus; Israeli BTV-5 strain ISR-2089/7/23 is signed by the inverted empty triangle; Israeli BTV-6 strain ISR-2095/3/17 is signed by the empty triangle; Israeli BTV-8 strain ISR-1741/15 is signed by the black square; Israeli BTV-8 strain ISR-2178/23 is signed by the empty square; Israeli BTV-11 strain 3265/2/21 is signed by the black triangle; Israeli BTV-12 strain ISR-2717/1/21 signed by the empty rhombus. The phylogeny was inferred using the Maximum Likelihood method and Tamura-Nei model method. Statistical support for nodes was obtained by bootstrapping (1,000 replicates); only values ≥ 70 % are shown. Scale bars indicate nucleotide substitutions per site. Viruses were identified by accession number/serotype/location/isolate/year.

Table S2. Information on closest identity of analyzed during the study recently identified Israeli bluetongue virus strains.

segment	serotype/year	local/old	local/new	N.Africa	S.Africa	Arab.peninsula	Europe	Com. Archipelago
		origin of most closest strains/%						
Seg-1	BTV-1 /2019	-	-	-	-	97.75/BTV-8	-	-
	BTV-1/2021	-	-	98.42-98.44/BTV-1		-	-	-
	BTV-3/2016-2020	-	-	-	96.34-96.80	-	-	-
	BTV-3/2020-2023	-	-	-	96.34-96.80	-	-	-
	BTV-3/2023*	-	-	-	96.34-96.80	-	-	-
	BTV-4/2015	-	-	-	-	-	-	-
	BTV-4/2023	98.55/BTV-5	-	-	-	-	-	-
	BTV-5/2023	-	-	-	-	-	98.04-98.35/BTV-3,-4	-
	BTV-6/2017	-	-	99.37-99.47/BTV-3	-	-	-	-
	BTV-8/2023	-	99.08/BTV-3	-	-	-	-	-
	BTV-9/2019	-	99.49/BTV-3	-	-	-	-	-
	BTV-11/2021	-	99.33-99.59/BTV-3	-	-	-	-	-
	BTV-12/2020	-	-	-	97.32%/BTV-4	-	-	-
Seg-3	BTV-1 /2019	-	-	-	98.05/BTV-5	-	-	-
	BTV-1/2021	-	-	-	-	-	97.49/BTV-4	-
	BTV-3/2016-2020	-	-	-	98.30-98.34/BTV-5	-	-	-
	BTV-3/2020-2023	-	99.29-99.49/BTV-3	-	-	-	-	-
	BTV-3/2023*	-	99.53/BTV-3	-	-	-	-	-
	BTV-4/2015	-	97.74/BTV-16	-	-	-	-	-
	BTV-4/2023	97.42-97.99/BTV-5,-8,-15	-	-	97.65/untyped	-	-	-
	BTV-5/2023	-	-	-	98.27/BTV-3,-16	-	-	-

	BTV-6/2017	-	-	96.98/BTV-4	-	-	-	-
	BTV-8/2023	-	96.86/BTV-3**	96.86/BTV-4	-	-	-	-
	BTV-9/2019	-	99.08/BTV-6	-	-	-	-	-
	BTV-11/2021	-	99.53/BTV-3	-	-	-	-	-
	BTV-12/2020	-	-	-	-	-	98.90/BTV-12	98.90/BTV-12
Seg-4	BTV-1 /2019	-	-	98.53/BTV-3	-	-	-	-
	BTV-1/2021	-	-	-	-	-	98.93/BTV-4	-
	BTV-3/2016-2020	-	-	-	-	-	97.72-97.87/BTV-12	-
	BTV-3/2020-2023	-	99.08-99.38/BTV-1	-	-	-	-	-
	BTV-3/2023*	-	99.64/BTV-12	-	-	-	-	-
	BTV-4/2015	99.63/BTV-2	-	-	-	-	-	-
	BTV-4/2023	-	-	98.37/BTV-3	-	-	-	-
	BTV-5/2023	-	-	98.17/BTV-2	-	-	-	-
	BTV-6/2017	-	-	99.33/BTV-3	-	-	-	-
	BTV-8/2023	-	99.59/BTV-1	-	-	-	-	-
	BTV-9/2019	-	-	98.47/BTV-2	-	-	-	-
	BTV-11/2021	98.83-98.93/BTV-24	-	-	-	-	-	-
	BTV-12/2020	-	-	-	-	-	-	98.93/BTV-12
Seg-5	BTV-1 /2019	-	-	-	-	-	98.24-98.61/BTV-3	-
	BTV-1/2021	-	-	-	97.98/BTV-24	-	-	-
	BTV-3/2016-2020	-	-	98.18-98.27/BTV-1	-	-	-	-
	BTV-3/2020-2023	-	99.37-99.77/BTV-1	-	-	-	98.24-98.61/BTV-3	-
	BTV-3/2023*	-	99.43/BTV-1	-	-	-	-	-
	BTV-4/2015	99.41/BTV-2	-	-	-	-	-	-
	BTV-4/2023	-	99.31/BTV-1	-	-	-	-	-
	BTV-5/2023	-	-	-	95.52/BTV-3	-	-	-
	BTV-6/2017	-	-	99.32/BTV-3	-	-	99.41/BTV-3	-

	BTV-8/2023	-	99.14/BTV-1	-	-	-	-	-
	BTV-9/2019	97.84/BTV-15	-	-	-	-	-	-
	BTV-11/2021	-	99.14/BTV-1	-	-	-	-	-
	BTV-12/2020	-	-	97.78/BTV-1	-	-	-	-
Seg-7	BTV-1 /2019	-	-	98.16/BTV-1	-	-	-	-
	BTV-1/2021	-	99.63/BTV-8	-	-	-	-	-
	BTV-3/2016-2020	-	-	97.87-98.08/BTV-3	-	-	-	-
	BTV-3/2020-2023	-	99.28-99.48/BTV-3	-	-	-	-	-
	BTV-3/2023*	-	99.30/BTV-3	-	-	-	-	-
	BTV-4/2015	99.30/BTV-24	-	-	-	-	-	-
	BTV-4/2023	-	-	-	98.70/BTV-1	-	-	-
	BTV-5/2023	-	98.08/BTV-3	-	-	-	-	-
	BTV-6/2017	-	-	-	-	-	99.02/BTV-3	-
	BTV-8/2023	-	99.55/BTV-8	-	-	-	-	-
	BTV-9/2019	-	99.73/BTV-4	-	-	-	-	-
	BTV-11/2021	99.32/BTV-8,-24	99.32/BTV-4	-	-	-	-	-
	BTV-12/2020	-	-	-	-	-	99.35/BTV-12	99.35/BTV-12
Seg-8	BTV-1 /2019	-	-	-	-	97.45/BTV-8	-	-
	BTV-1/2021	-	-	-	98.18/BTV-4	-	-	-
	BTV-3/2016-2020	-	-	97.42-97.59/BTV-1	-	-	-	-
	BTV-3/2020-2023	-	-	97.21-97.58/BTV-1				
	BTV-3/2023*	-	99.37-99.64/BTV-4,-9	-	-	-	-	-
	BTV-4/2015	99.56/BTV-2	-	-	-	-	-	-
	BTV-4/2023	-	98.06/BTV-3	-	-	-	-	-
	BTV-5/2023	-	-	-	-	-	98.65/BTV-4	-
	BTV-6/2017	-	-	-	98.57/BTV-4	-	-	-

	BTV-8/2023	-	98.45/BTV-1	-	-	-	-	-
	BTV-9/2019	99.64/BTV-2	-	-	-	-	-	-
	BTV-11/2021	-	99.35/BTV-3	-	-	-	-	-
	BTV-12/2020	-	-	-	-	-	-	98.66/BTV-12
Seg-9	BTV-1 /2019	-	-	98.95/BTV-2	-	-	-	-
	BTV-1/2021	99.39/BTV-24	-	-	-	-	-	-
	BTV-3/2016-2020		98.56-98.86/BTV-3	-	-	-	-	-
	BTV-3/2020-2023	99.41/BTV-6	-	-	98.91/BTV-1	-	-	-
	BTV-3/2023*	98.94/BTV-24	-	-	-	-	-	-
	BTV-4/2015	-	-	-	-	-	-	-
	BTV-4/2023	97.68/BTV-15	-	-	-	-	-	-
	BTV-5/2023	-	-	-	96.28/BTV-24	-	-	-
	BTV-6/2017	-	-	-	-	-	99.12/BTV-3	-
	BTV-8/2023	-	98.95/BTV-3	-	-	-	-	-
	BTV-9/2019	-	-	-	-	-	97.68/BTV-8	-
	BTV-11/2021	-	99.13/BTV-3	-	-	-	-	-
	BTV-12/2020	-	-	-	-	-	98.36/BTV-12	98.73/BTV-12
Seg-10	BTV-1 /2019	-	-	-	99.15/BTV-3	-	-	-
	BTV-1/2021	-	-	-	99.47/BTV-7	-	-	-
	BTV-3/2016-2020	-	-	-	-	-	97.06-97.32/BTV-8	-
	BTV-3/2020-2023	99.24-99.51/BTV-3	-	-	-	-	-	-
	BTV-3/2023*	99.25-99.50/BTV-3	-	-	-	-	-	-
	BTV-4/2015	98.86-99.49/BTV-4,-24	-	-	-	-	-	-
	BTV-4/2023	99.48/BTV-24	-	-	-	-	-	-
	BTV-5/2023	-	-	-	99.85/BTV-5	-	-	-
	BTV-6/2017	-	-	-	-	-	-	97.82/BTV-12
	BTV-8/2023	99.36/BTV-24	-	-	-	-	-	-

	BTV-9/2019	-	99.74/BTV-untyped	-	-	-	-	-
	BTV-11/2021	99.36/BTV-24	-	-	-	-	-	-
	BTV-12/2020	-	-	-	-	-	-	99.36/BTV-12
total		20	32	17	18	2	15	7

local/old- Israeli bluetongue strains identified before 2013. local/new- Israeli bluetongue strains identified before 2013. N. Africa- North Africa. S. Africa- South Africa. Arab.peninsula- Arabian Peninsula. Com. Archipelago- Comoros Archipelago.
